# Methods of Increasing the Performance of Radionuclide Generators Used in Nuclear Medicine: Daughter Nuclide Build-Up Optimisation, Elution-Purification-Concentration Integration, and Effective Control of Radionuclidic Purity

**DOI:** 10.3390/molecules19067714

**Published:** 2014-06-10

**Authors:** Van So Le, Zoe Phuc-Hien Do, Minh Khoi Le, Vicki Le, Natalie Nha-Truc Le

**Affiliations:** 1 Cyclopharm Ltd., Sydney 2234, NSW, Australia; 2 Medical Isotope Techniques (MEDISOTEC), 14(1) Dwyer Street, Gymea 2227, NSW, Australia; E-Mails: sohien_tvk@yahoo.com (Z.P.-H.D.); minh.kh.le@gmail.com (M.K.L.); vi_3001@hotmail.com (V.L.); natalie.nt.dubs@gmail.com (N.N.-T.L.)

**Keywords:** radionuclide generator, radioisotope concentrator, mean progress function, optimal build-up time, effective specific activity, radionuclidic purity, nuclear medicine, radiopharmaceutical, ^99m^Tc, ^68^Ga

## Abstract

Methods of increasing the performance of radionuclide generators used in nuclear medicine radiotherapy and SPECT/PET imaging were developed and detailed for ^99^Mo/^99m^Tc and ^68^Ge/^68^Ga radionuclide generators as the cases. Optimisation methods of the daughter nuclide build-up *versus* stand-by time and/or specific activity using mean progress functions were developed for increasing the performance of radionuclide generators. As a result of this optimisation, the separation of the daughter nuclide from its parent one should be performed at a defined optimal time to avoid the deterioration in specific activity of the daughter nuclide and wasting stand-by time of the generator, while the daughter nuclide yield is maintained to a reasonably high extent. A new characteristic parameter of the formation-decay kinetics of parent/daughter nuclide system was found and effectively used in the practice of the generator production and utilisation. A method of “early elution schedule” was also developed for increasing the daughter nuclide production yield and specific radioactivity, thus saving the cost of the generator and improving the quality of the daughter radionuclide solution. These newly developed optimisation methods in combination with an integrated elution-purification-concentration system of radionuclide generators recently developed is the most suitable way to operate the generator effectively on the basis of economic use and improvement of purposely suitable quality and specific activity of the produced daughter radionuclides. All these features benefit the economic use of the generator, the improved quality of labelling/scan, and the lowered cost of nuclear medicine procedure. Besides, a new method of quality control protocol set-up for post-delivery test of radionuclidic purity has been developed based on the relationship between gamma ray spectrometric detection limit, required limit of impure radionuclide activity and its measurement certainty with respect to optimising decay/measurement time and product sample activity used for QC quality control. The optimisation ensures a certainty of measurement of the specific impure radionuclide and avoids wasting the useful amount of valuable purified/concentrated daughter nuclide product. This process is important for the spectrometric measurement of very low activity of impure radionuclide contamination in the radioisotope products of much higher activity used in medical imaging and targeted radiotherapy.

## 1. Introduction

Today most radionuclide generators are used for nuclear medicine purposes. The generators routinely used in daily nuclear medicine diagnosis and therapeutic treatment are ^99^Mo/^99m^Tc, ^113^Sn/^113m^In, ^81^Rb/^81m^Kr, ^82^Sr/^82^Rb, ^68^Ge/^68^Ga, ^62^Zn/^62^Cu, ^188^W/^188^Re, ^9^°Sr/^9^°Y, and ^166^Dy/^166^Ho among which the ^99^Mo/^99m^Tc generator is used most effectively. Other generator systems are used to some extent or are under development. More than 80% of diagnostic imaging procedures of nuclear medicine based on ^99m^Tc-radiopharmaceuticals are performed world-wide every year. The short-lived gamma or positron emitting radioisotopes produced from radionuclide generators are effectively used in diagnostic nuclear medicine and in biomedical research, while the generator-produced long-lived beta- and alpha-emitting radioisotopes are used in radiotherapy. The ^99m^Tc generator is a well-known example and most widely used in single-photon emission computed tomography (SPECT) and the ^68^Ga and ^82^Rb in positron emission tomography (PET) for diagnostic imaging. Expansion of the use of radionuclides produced from radionuclide generators is always desirable, subsequently benefiting superiority of the SPECT and PET-based molecular imaging technique. Usually, a radionuclide generator, or colloquially a “radioisotope cow” is a device used to extract the short-lived daughter nuclide generated from the radioactive decay of long-lived parent nuclide. As such, it can be easily transported over long distances to radiopharmacies where its decay product daughter radionuclide is extracted for daily use. The safe utilisation of the nuclide generators is definitely controlled by the quality factors required by the health authorities. However, the acceptability of a radionuclide generator to be used in nuclear diagnostic/therapeutic procedures and the quality of SPECT imaging diagnosis and/or endo-radiotherapy are controlled by the generator design and its operation management/“daughter nuclide milking” schedule. The efficacy of a radionuclide generator used in nuclear medicine depends on the concentration and specific radioactivity (*SA*) of the daughter nuclide in the solution produced from the generator, because the volume and the mass of bio-medically active radiolabelled agents used in one given injection dose of daughter nuclide-labelled radiopharmaceuticals are limited to avoid any possible side/adverse effect for the metabolic system of the patient body. Daughter nuclide concentration is determined by the radioactivity yield of each “milking”/elution of the generator and the final volume of the daughter nuclide solution. This solution volume is fixed and defined by the generator design which is dictated by a given radiochemical processing process. However, the specific radioactivity of (*SA*) of the daughter nuclide is affected by the “milking” schedule only.

Additionally, the cost-effective utilisation of the generator is controlled by the daughter nuclide yield obtained for the whole lifetime of a given generator, which is the sum of yields obtained in each “milking” (elution/separation) and is determined by the “milking” schedule (the daughter nuclide build-up time schedule) of the generator. In conclusion, for an established design of generator system, the build-up time schedule of the daughter nuclide for the generator operation management will determine not only the concentration and specific radioactivity of the daughter nuclide solution obtained from the generator but also the cost-effective utilisation of the generator system. Thus the effective utilisation of the generator is experienced as a result of proper “milking” management based on the optimal schedule of the daughter nuclide build-up time. Similarly, the optimisation in the daughter nuclide decay time schedule *versus* radioactivity of the sample for a radionuclidic purity test is also addressed for the optimal management of the generator utilisation. Therefore the report on this issue is also included in this article.

With the success in the development of the integrated elution-purification-concentration systems (RADIGIS-Radioisotope Generator Integrated System) and the radioisotope concentrator device ULTRALUTE^®^ to achieve a small volume (~1.0 mL) of the product solution of daughter nuclide obtained from the variable design of the generator systems as reported in our previous papers [1,2,3,4,5,6,7,8,9,10], the optimisation methods of radionuclide generator operation management as mentioned above will in turn become the most important subject to be discussed in the series of our generator development projects.

It is realized that no report on the optimisation of daughter nuclide build-up time (stand-by time of the generator for each “milking”) for the generator operation have been available in the literature until now. Our present work is focused on providing the optimisation methods first time developed for the radionuclide generator operation management to increase the effectiveness of the radionuclide generator utilisation.

## 2. Theoretical Approach and Method Development

### 2.1. Daughter Nuclide Build-Up Optimisation for Improvement of Production Yield and Specific Radioactivity

*General consideration*: For the reason of ensuring a convenient and effective radiochemical separation, the following general radionuclide decay scheme is usually used in the practice of radionuclide generator production:

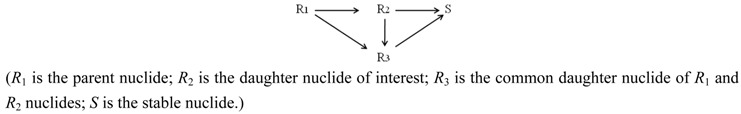



Radioactivity build-up (*A*_2_) of the daughter nuclide *R*_2_ of interest in the generator:

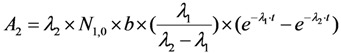
(1)
(*λ*_1_ and *λ*_2_ are the decay constants of the parent and daughter nuclides, respectively; *N*_1,0_ is the atom numbers of the parent nuclide at the build-up time time *t* = 0; *b* is the branch decay ratio of the parent nuclide *R*_1_ leading to the daughter nuclide *R*_2_.)

The maximal build-up time *t*_max_ for nuclide *R*_2_ (at which the maximal activity build-up (yield) of the *R*_2_ nuclide in the generator is available):

*t*_max_ = [ln(*λ*_2_ / *λ*_1_)] / (*λ*_2_ − *λ*_1_) (2)

**Figure 1:**
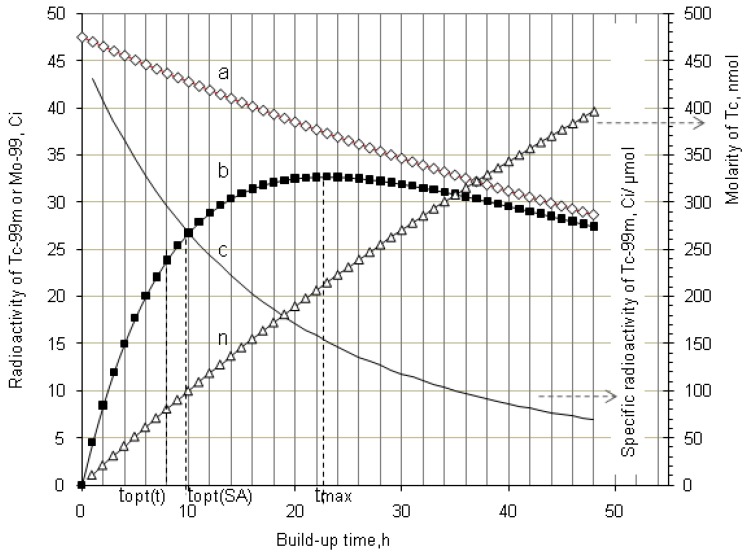
^99m^Tc build-up in the ^99^Mo/^99m^Tc generator system: ^99^Mo activity (a); ^99m^Tc activity (b); Specific radioactivity of ^99m^Tc (c); Total atom numbers *N* or molarities of Tc (n); *t*_max_ is the maximal build-up time for nuclide ^99m^Tc; *t_opt_*_(*t*)_ and *t_opt_*_(*SA*)_ are the optimal build-up time values calculated in the optimisation process of the radioactivity build-up *versus* standby time and/or *versus* specific activity of the daughter nuclide ^99m^Tc, respectively (Section 2.1.1 and Section 2.1.2; Table 1).

**Figure 2:**
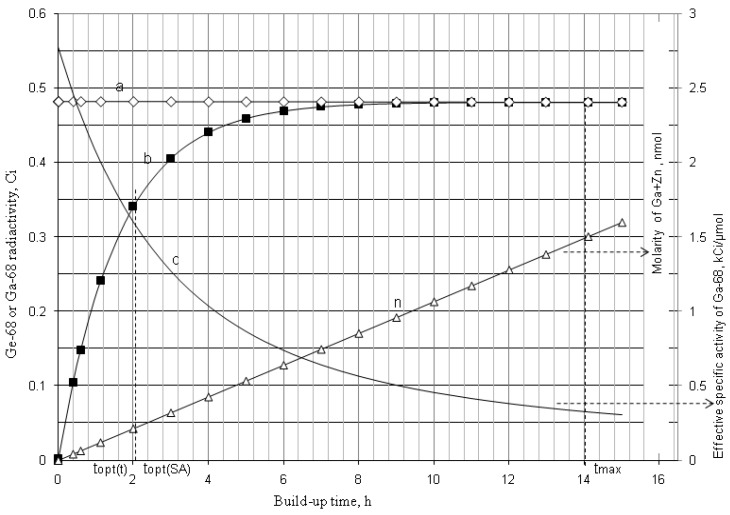
^68^Ga build-up in the ^68^Ge/^68^Ga generator system: ^68^Ge activity (a); ^68^Ga activity (b); Effective specific radioactivity of ^68^Ga (c); Total atom numbers *N* or molarities of Ga +Zn (n); *t*_max_ is the maximal build-up time for nuclide ^68^Ga; *t_opt_*_(*t*)_ and *t_opt_*_(*SA*)_ are the optimal build-up time values calculated in the optimisation process of the radioactivity build-up *versus* standby time and/or *versus* specific activity of the daughter nuclide ^68^Ga, respectively (Section 2.1.1 and Section 2.1.2; Table 1).

As shown in Figure 1 and Figure 2, it is the fact that at the start of the *R_i_* build-up the convex exponential increase of radioactivity *A*_2_ is faster than the linear increase of build-up time *t*. However, the increase of the value *A*_2_ will slow down after a certain time period. This relationship can be used for an optimisation of the radioactivity build-up *versus* build-up time (or the standby time) of the daughter nuclide *R*_2_ in the generator, which will be formulated in Section 2.1.1, Section 2.1.2, and Section 2.1.3.

*Specific radioactivity definitions used for optimisation assessment*: The followings can be justified based on the above general radionuclide decay scheme:

- If *R*_2_ ≠ *R*_3_ ≠ *S* (*R*_2_, *R*_3_ and *S* are the nuclides of different chemical elements) then *R*_2_ and *R*_3_ are radionuclides available in a carrier-free state and their elemental specific radioactivity (*SA*) is invariable for all the time. In this case, the *SA* value can be evaluated without acknowledgement of the atom numbers of given nuclide using the following equation [11].
  *SA*_Carrier-free_ = 6.022 × 10^23^ × *λ*_Ri_ (Bq/Mol) (3)
- In the case of *R*_2_ ≡ *R*_3_ ≠ *S* (*R*_2_ and *R*_3_ are the radionuclides of the same chemical element; *S* is a stable nuclide of another element), both the *R*_2_ and *R*_3_ radionuclides are available in a carrier-included state and their elemental specific radioactivity is variable with the build-up time (^99^Mo/^99m^Tc system shown below as an example). With an assumption of ignoring the insignificant amount of stable nuclide *S* formed in the system, the atom numbers (*N*) of all daughter radionuclides *R*_2_ and *R*_3_ generated from the parent nuclide *R*_1_ is
  *N* = *N*_*R*_2__ + *N*_*R*_3__ = *N*_1,0_ − *N*_1_ = *N*_1,0_ × (1 − *e*^−*λ*_1_*·t*^)
  This *N* value will be used for the calculation of elemental specific radioactivity in the following process of optimisation assessment.
- In the case of *R*_2_ ≈ *R*_3_ ≈ *S* (*R*_2_, *R*_3_ and *S* are the isotopes of different chemical elements which have a similar chemical property of interest for a specific application such as the coordinative radiolabelling of radiopharmaceuticals, the *R*_2_ and *R*_3_ radionuclides are available in a non-elemental carrier-included state and the specific radioactivity of radionuclide *R*_2_, named as “*Effective Specific Radioactivity*” (*ESA*), is variable with the build-up time (^68^Ge/^68^Ga system shown below as an example). The effective specific radioactivity is conveniently defined as the radioactivity of a specified radioactive daughter nuclide (*R*_2_ or *R*_3_) per the total atom numbers of three nuclides *R*_2_, *R*_3_, and *S*. The atom numbers (*N*) of all related daughter nuclides which are generated from the parent nuclide *R*_1_ is
  *N* = *N*_*R*_2__ + *N*_*R*_3__ + *N_S_* = *N*_1,0_ − *N*_1_ = *N*_1,0_ × (1 − *e*^−*λ*_1_*·t*^)
  This *N* value will also be used for the calculation of *ESA* values in the following process of optimisation assessment.

As shown in the two last cases above, both values of *SA* and *ESA* of the carrier-included radionuclide *R_i_* can be calculated using the same following equation:

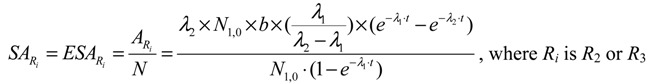
(4)

As shown in Figure 1 and Figure 2, it is the fact that at the start of the *Ri* build-up the total atom numbers *N* (or molarities) of three daughter nuclides *R*_2_, *R*_3_, and *S* will increase convexly slower than the exponentially increasing activity *A*_2_ of the daughter nuclide *R*_2_. However, the value of *N* (or molarities) will increase faster than that of *A*_2_ after a certain time period, because the increase of *N* value (or molarities) is only affected by the decay of the parent nuclide *R*_1_, while *A*_2_ value is controlled by both the decays of the parent and daughter nuclides. This relationship can be used for an optimisation of the radioactivity build-up *versus* *N* value (or molarities) of the daughter nuclide *R*_2_. Taking into account the above mentioned Equation (4) for the calculation of *SA* and *ESA* values, the mean progress function for optimisation of the daughter nuclide build-up *versus* specific activity will be formulated in Section 2.1.2.

The following examples are typical decay schemes used in the radionuclide generators of practical application. They are used for demonstration of the optimisation methods developed in this report.

*^99^Mo/^99m^Tc system*: The decay scheme of ^99^Mo/^99m^Tc system used in the ^99m^Tc-generator production processes is presented as follows:

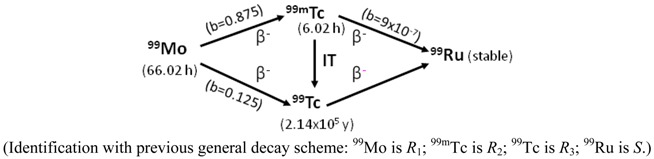



As shown, the formation of ^99^Ru stable nuclides is insignificant due to the small branch-decay factors of the ^99m^Tc (*b* = 9 × 10^−7^) and long lived ^99^Tc nuclides to form ^99^Ru nuclide. This scheme shows that ^99m^Tc is in a carrier-included state and the total numbers of Tc-nuclides at the build-up time *t* is described as follows:

*N_Tc_* = *N_Tc_*_−99_ + *N_Tc_*_−99*m*_ = *N_Mo_*_−99,0_ − *N_Mo_*_−99_ = *N_Mo_*_−99,0_ × (1 − *e*^−*λ*_*Mo*−99_·*t*^) (5)

The elemental specific activity of carrier-included ^99m^Tc in the ^99m^Tc generator system or in the ^99m^Tc-eluate is calculated by a combination of Equations (1) and (5) as follows:

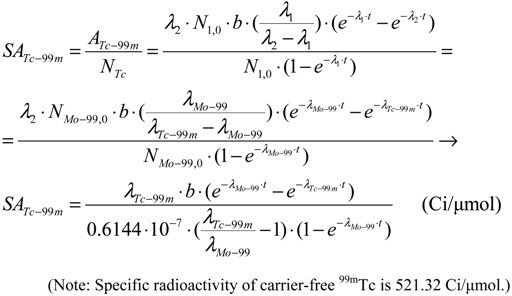
(6)


*^68^Ge^/68^Ga system*: The decay scheme of ^68^Ge^/68^Ga system used in the ^68^Ga-generator production processes is presented as follows.


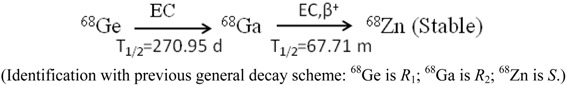


This scheme shows that ^68^Ga is in a carrier-free state and its specific radioactivity is a constant value (2776.4 Ci/μmol, calculated using Equation (3). 

As shown in the ^68^Ge/^68^Ga decay scheme, ^68^Ga decays to ^68^Zn relatively rapidly. There is a defined amount of ^68^Zn^2+^ ions that accumulate on the sorbent and then can possibly be eluted into the ^68^Ga eluate during the generator elution operation. Zn^2+^ ions have the coordination chemistry property similar to ^68^Ga^3+^ ions in the reaction of ^68^Ga-radiolabelling with macrocyclic ligands in the targeting radiopharmaceutical preparation process, such as the preparation of radiopharmaceuticals ^68^Ga-OTATATE, ^68^Ga-DOTATOC, and ^68^Ga-DOTANOC for tumour imaging [12]. So the presence of Zn^2+^ ions in the ^68^Ga solution will definitely influence the ^68^Ga–labelling yield of ^68^Ga-radiopharmaceuticals. Due to the similarity in the incorporation into labelled compounds between ^68^Zn and ^68^Ga, the impact on the radioisotope dilution of the ^68^Ga atoms with ^68^Zn atoms is considered thus much as with the non-radioactive Ga atoms which would be present in the ^68^Ga solution. In this case the use of *ESA* value of the ^68^Ga-solution will be useful and all related radiochemical assessments should be performed with *ESA* value instead of the elemental specific radioactivity (*SA*) value of the carrier-free ^68^Ga solution. In this case *ESA* value is calculated using Equation (4) as clarified above.

The total numbers of ^68^Ga and ^68^Zn nuclides at the build-up time t is described as follows:

*N* = *N_Ga_*_−68_ + *N_Zn_*_−68_ = *N_Ge_*_−68,0_ − *N_Ge_*_−68_ = *N_Ge_*_−68,0_ · (1 − *e*^−*λ*_*Ge*−68_·*t*^) (7)

The *ESA* value of ^68^Ga nuclide is calculated as follows:

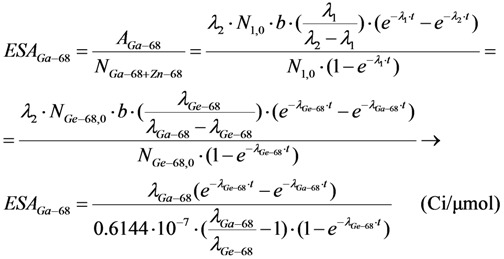
(8)

#### 2.1.1. Mean Progress Function for Optimisation of the Daughter Nuclide Build-up *versus* Buildup Time (or Standby Time)

This function is formulated based on the fact that at the start of the daughter nuclide build-up the convex exponential increase of the activity *A*_2_ of daughter nuclide *R*_2_ is faster than the linear increase of the build-up time *t*.

However, *A*_2_ value will increase more slowly than *t* value after a certain time period. The mean progress function for optimisation of the daughter nuclide build-up *versus* stand-by time is formulated as follows.

*Notation: f*(*A*,*t*) is the mean progress function for optimisation of the daughter nuclide build-up *versus* stand-by time *t*. *t_opt_*_(*t*)_ is the optimal build-up time for the daughter nuclide build-up *versus* stand-by time; *A* = *A*_2_ is the build-up radioactivity of the daughter nuclide *R*_2_ and other notations are the same as in Equation (1).

The meaning of this mean progress function is that the progressive increase in the daughter nuclide activity build-up is related to the build-up time progress needed for increasing one unit of the daughter nuclide build-up activity on average for the build-up time period *t*.

In other words, the progressive increase in the daughter nuclide activity build-up is compared with the quotient of the build-up time increase per unit of daughter nuclide build-up activity:

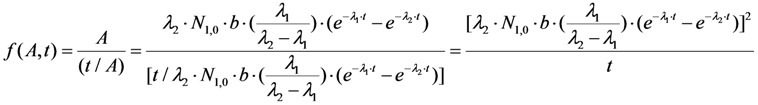
(9)

To find the stationary point (maximum point) of this function, we differentiate, set the derivative equal to zero and solve the equation to find out the time value *t*_max,*f*(*A*,*t*)_ at which the value of the function *f*(*A*,*t*) reaches the maximum:

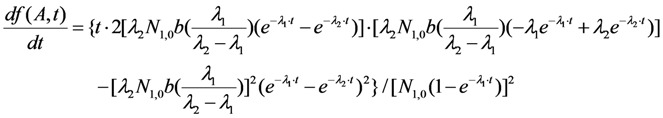
(10a)

At the time point *t* = *t*_max,*f*(*A*,*t*)_ the derivative of the *f*(*A*,*t*) function is equal to zero:

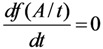


From the Equation (10a), by replacing *t* = *t*_max,*f*(*A*,*t*)_ it is re-written as follows:

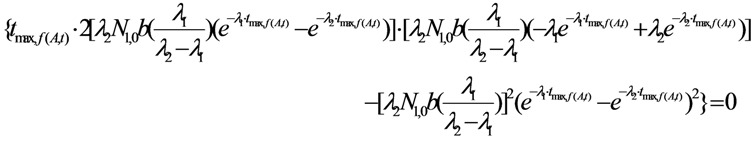



Re-arranging this equation, the followings are obtained:

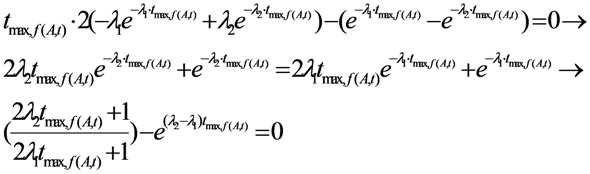
(10b)

Finally, the time value *t*_max,*f*(*A*,*t*)_ will be obtained as a result of the solution of Equation (10b).

As clarified by the meaning of the *f*(*A*,*t*) function described above, it is stated that the time value *t*_max,*f*(*A*,*t*)_ is the optimal build-up time *t_opt_*_(*t*)_ of the daughter nuclide build-up activity (*A*) *versus* the build-up time *t*, or *t*_max,*f*(A,*t*)_ = *t_opt_*_(*t*)_. By replacing the value *t_opt_*_(*t*)_ into Equation (10b), the following is obtained:

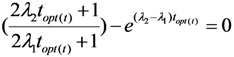
(11)

This identification/attribution is based on the fact that the function of daughter nuclide build-up activity will develop from the fast growing state with the increasing values of function *f*(*A*,*t*) = *A*/(*t*/*A*) to the slow-down state with the decreasing *f*(*A*,*t*) = *A*/(*t*/*A*) values via a transient point *A_opt_*_(*t*)_ (so-called optimal build-up activity) achievable at the optimal build-up time point *t_opt_*_(*t*)_.

**Figure 3:**
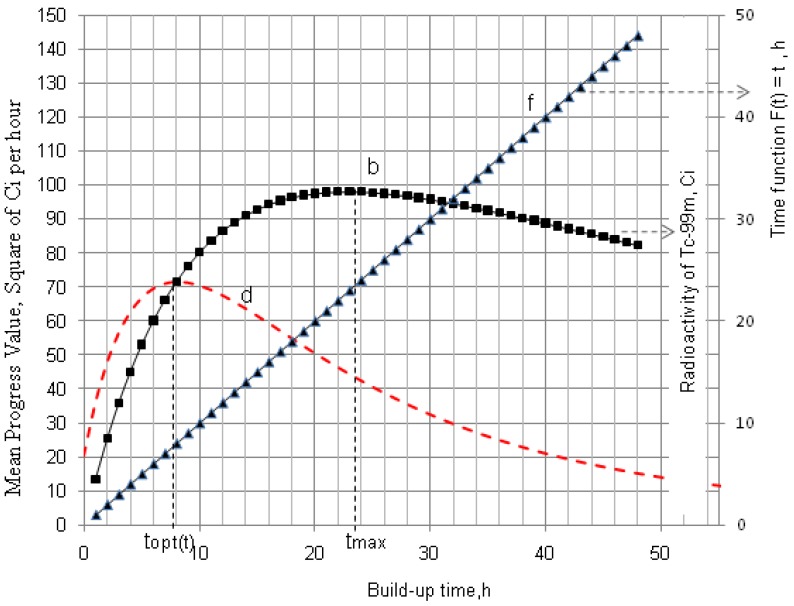
^99m^Tc build-up optimisation of ^99^Mo/^99m^Tc generator system: b, ^99m^Tc activity build-up (Referred to curve b of Figure 1); d, Mean progress function of ^99m^Tc-build-up *versus* stand-by time; f is the time function *f*(*t*) = *t*. *t*_max_ is the maximal build-up time for nuclide ^99m^Tc; *t_opt_*_(*t*)_ is the optimal time value calculated with Equation (11) in the optimisation process of the radioactivity build-up *versus* standby time of the daughter nuclide ^99m^Tc.

As an explanatory example, the ^99m^Tc-build-up optimisation of ^99^Mo/^99m^Tc generator system is shown in Figure 3. The *t_opt_*_(*t*)_ values of 50 parent/daughter nuclide pairs calculated using Equation (11) are reported in the section “Results and Discussions” (Section 4.1 and Table 1).

#### 2.1.2. Mean Progress Function for Optimisation of the Daughter Nuclide Build-up *versus* Specific Activity

Taking into account the above mentioned formulations for the specific activity values *SA* and *ESA*, the mean progress function for optimisation of the daughter nuclide build-up *versus* specific activity is formulated based on the fact that at the start of the daughter nuclide build-up, the total atom numbers (*N* = *N*_1.0_(1 − *e*^−*λ*_1_*t*^) of the daughter nuclides *R*_2_, *R*_3_, and *S* will increase slower than the activity *A*_2_ of the daughter nuclide *R*_2_. However, the atom number value *N* of the daughter nuclides will increase faster than the value *A*_2_ after a certain time period, because *N* value is only affected by the decay of the parent nuclide *R*_1_, while *A*_2_ value is controlled by both the decays of the parent and daughter nuclides. The mean progress function for optimisation of the daughter nuclide build-up *versus* specific radioactivity is formulated as follows:

*Notation*: *f*(*A*,*SA*) is the mean progress function for optimisation of the daughter nuclide build-up *versus* specific radioactivity. *t_opt_*_(*SA*)_ is the optimal build-up time for the daughter nuclide build-up *versus* specific radioactivity; *N* is the nuclide atom numbers as clarified in Section 2.1; *A* = *A*_2_ is the build-up radioactivity of the daughter nuclide *R*_2_ and other notations are the same as in Equation (1).

The meaning of this mean progress function is that the progressive increase in the daughter nuclide activity build-up is related to the total daughter nuclide atom numbers grow-up needed for increasing one unit of the daughter nuclide build-up activity on average for the build-up time period *t*. 

In other words, the progressive increase in the daughter nuclide activity build-up is compared with the quotient of the total daughter nuclide atom number increase per unit of daughter nuclide build-up activity:

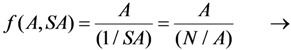


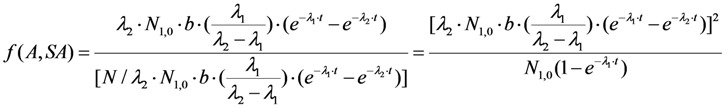
(12)

To find the stationary point (maximum point) of this function, we differentiate, set the derivative equal to zero and solve the equation to find out the time value *t*_max,*f*(*A*,*SA*)_ at which the value of the function *f*(*A*,*SA*) reaches the maximum:

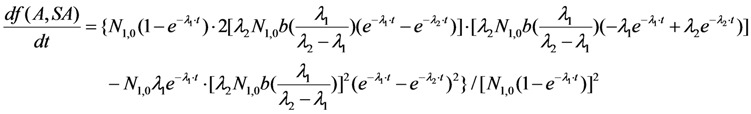
(13a)

At the time point *t* = *t*_max,*f*(*A*,*SA*)_ the derivative of the *f*(*A*,*SA*) function is equal to zero:

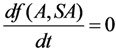


From Equation (13a), by replacing *t* = *t*_max,*f*(*A*,*SA*)_ it is re-written as follows:

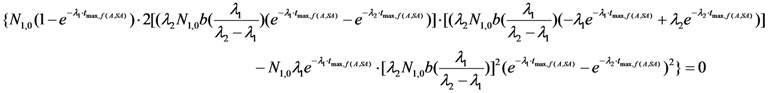


Re-arranging this equation, the following are obtained:

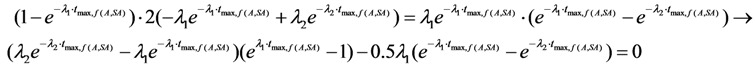
(13b)

Finally, the time value *t*_max,*f*(*A*,*SA*)_ will be obtained as a result of the solution of Equation (13b).

As clarified by the meaning of the *f*(*A*,*SA*) function described above, it is stated that the time value *t*_max,*f*(*A*,*SA*)_ is the optimal build-up time *t_opt_*_(*SA*)_ of the daughter nuclide build-up activity (*A*) *versus* specific activity *SA*, or *t*_max,*f*(*A*,*SA*)_ = *t_opt_*_(*SA*)_. 

By replacing the value *t_opt_*_(*SA*)_ into Equation (13b), the following is obtained:

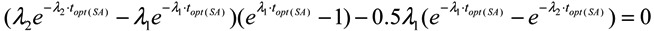
(14)

This identification/attribution is based on the fact that the function of daughter nuclide build-up activity will develop from the fast growing state with the increasing values of function *f*(*A*,*SA*) = *A*/(*N*/*A*) to the slow-down state with the decreasing *f*(*A*,*SA*) = *A*/(*N*/*A*) values via a transient point *A_opt_*_(*SA*)_ (so-called optimal build-up activity) achievable at the optimal build-up time point *t_opt_*_(*SA*)_. 

**Figure 4:**
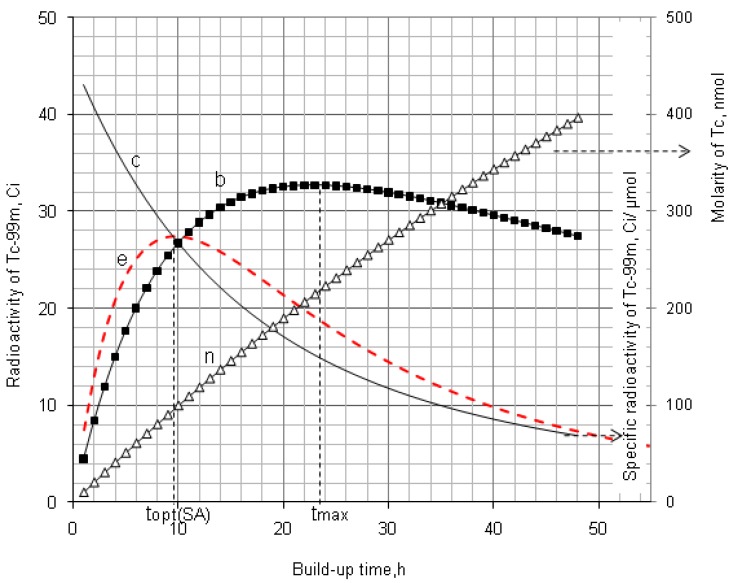
^99m^Tc build-up optimisation of ^99^Mo/^99m^Tc generator system: b, ^99m^Tc activity build-up (Referred to curve b of Figure 1); c, Specific radioactivity of ^99m^Tc (Referred to curve c of Figure 1); n, the total atom numbers *N* or molarities of Tc; e, Mean progress function of the ^99m^Tc-build-up *versus* specific activity of ^99m^Tc; *t*_max_ is the maximal build-up time for nuclide ^99m^Tc; *t_opt_*_(*SA*)_ is the optimal time value calculated in the optimisation process of the radioactivity build-up *versus* specific activity of the daughter nuclide ^99m^Tc.

As an explanatory example, the ^99m^Tc- build-up optimisation of ^99^Mo/^99m^Tc generator system is shown in Figure 4. The t_opt__(SA__)_ values of 50 parent/daughter nuclide pairs calculated using Equation (14) are reported in the section “Results and Discussions” (Section 4.1 and Table 1). (*Note*: In the case of carrier-free daughter radionuclides, the atom numbers value *N* is decreased during increased build-up of the daughter nuclide due to its decay, so, no mean progress function *f*(*A*,*SA*) will exist).

#### 2.1.3. Method of Early Elution Schedule for Increasing the Daughter Nuclide Production Yield and Effective Utilisation of Parent Nuclide

The daughter nuclide activity yield of the radionuclide generator can be increased by performing an optimal regime of multiple “early” elutions (the generator is more frequently eluted) which are performed at any time before maximal build-up of the daughter nuclide. This idea has been proved in our previous papers [5,7,10] which reported the method for evaluation of the effectiveness of “early” elution regime in comparison with a single elution performed at maximal build-up time for the ^99m^Tc-generator system. Actually, this is a general method which can be used for all radionuclide generators using different parent/daughter nuclide pairs. For general application of this method, the daughter nuclide-yield ratio (*R_y_*) is set up and a general equation of *R_y_* value assessment for the radionuclide generator systems is derived. *R_y_* is defined as a quotient of the sum of daughter nuclide build-up activity (or elution yields) eluted in all *i* “early” elutions divided by the maximal daughter nuclide build-up activity *A*_2(*max*)_ (or elution yield) which would be eluted from the generator at maximal build-up time *t*_max_:

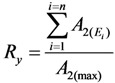
(15)
where *A*_2(*E_i_*)_ and *A*_2(*max*)_ are the daughter nuclide build-up activities (or elution yields) at the elution time *t*_(*E_i_*)_ and *t*_max_, respectively (*E_i_* is indexed for the *i*th elution).

The build-up time period (*t_b_*) for each “early” elution is given as *t_b_* = (*t*_max_/*i*), where *i* is the integer number of the “early” elutions. 

*Daughter nuclide-yield ratio (R_y_) calculation for a multiple “early” elution schedule*: The total daughter nuclide-elution yields eluted in all *i* elutions is the sum of the daughter nuclide -activities (*A*_2(*Ei*)_) achieved at different “early” elution *i*. The equation for evaluation of this amount is derived from Equation (1) and described as follows:

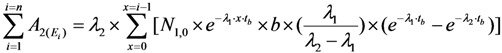
(16)

The maximal daughter nuclide-activity build-up/yield of the radionuclide generator is described using Equations (1) and (2) as follows:

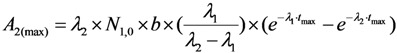
(17)

The daughter nuclide-yield ratio (*R_y_*) derived from Equations (16) and (17) is the following:

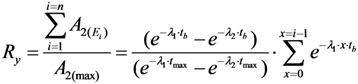
(18)
where, *i* is the number of the early elutions needed for a practical schedule of the generator elution, as an example, planned for a series of consecutive imaging scans. The build-up time (*t_b_*) for each “early” elution is determined *t_b_* = (*t*_max_/*i*). *x* is the number of the elutions which have been performed before starting a daughter nuclide-build-up process for a consecutive elution. At this starting build-up time point it is assumed that no residual daughter nuclide atoms have been left in the generator from a preceding elution (*i.e.*, the daughter nuclide-elution yield of the preceding elution is assumed 100%). 

**Figure 5:**
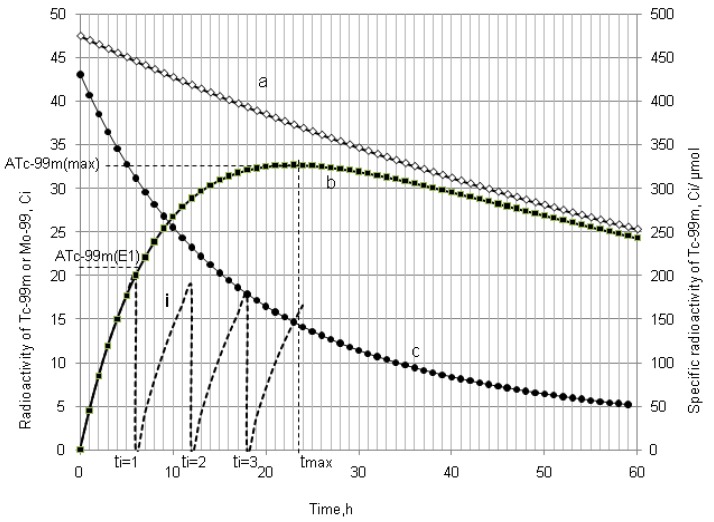
Kinetics of radioactive decay/^99m^Tc-activity build-up in the generator eluted with an early elution schedule: a, ^99^Mo-activity; b, ^99m^Tc-activity build-up from beginning; i, ^99m^Tc-activity growth/eluted at 6-h elutions; c, Specific Activity of ^99m^Tc in the system of ^99m^Tc-radioactivity build-up from beginning (the detailed discussion is available in Section 4.1.2).

**Figure 6:**
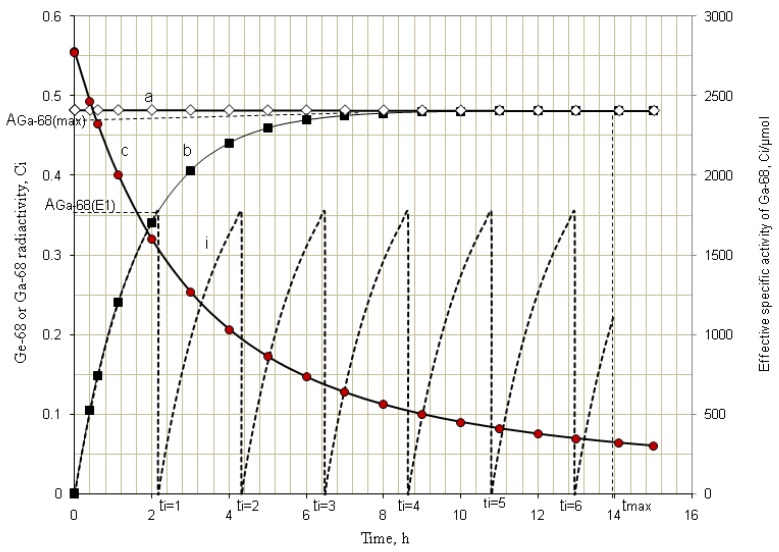
Kinetics of radioactive decay/^68^Ga-activity build-up in the generator eluted with an early elution regime: a, ^68^Ge-activity; b, ^68^Ga-activity build-up from beginning; i, ^68^Ga-activity growth/eluted at 2.1-h elutions; c, Effective Specific Activity of ^68^Ga in the system of ^68^Ga-radioactivity build-up from beginning (the detailed discussion is available in Section 4.1.2).

The examples of ^99^Mo/^99m^Tc and ^68^Ge/^68^Ga systems for the demonstration of method developed are shown in Figure 5 and Figure 6, respectively. The *R_y_* values of the early elution schedule for these systems are evaluated and reported in Section 4.1.2 (Results and Discussion).

### 2.2. Method of Radioisotope Concentrator Design for Use in the Optimisation of Generator Elution to Increase the Performance of Radionuclide Generators: Concentrating ^99m^Tc Solution Eluted from ^99^Mo/^99m^Tc Generator as a Case

The radioisotope concentration process not only has positive impact on the extension of useful generator lifetime, but also is capable to increase the effectiveness of ^99m^Tc and ^99^Mo utilisation by performing consecutive early elutions of the generator at any time before maximal build-up of the ^99m^Tc daughter nuclide. The ^99m^Tc activity yield of the generator can be increased by performing an optimal regime of multiple consecutive “early” elutions (the generator is more frequently eluted) combined with a process of ^99m^Tc-eluate concentration. We have developed a method of assessment of concentration factor values for the design of radioisotope concentrators. This method relies on the basic parameters currently used in the chromatographic processes such as the retention time/volume and the distribution coefficient of the solute [5,6]. This evaluation is an important guide for designing the concentrator with optimal operation conditions. A standardization method of concentration factor evaluation is to use an elution performed with normal saline solution (0.9% NaCl) as a reference. In this case, the normal saline plays both the role of a generator eluate containing solute (^99m^Tc), which is fed/loaded onto the following concentration column to be concentrated and that of the eluate of final concentrated ^99m^Tc-product saline solution which is stripped from the concentration column. This approach is also useful for the evaluation of the effectiveness of one concentration process (Sorbent-eluent system) in comparison with other concentration systems which could or would be performed under similar (normalized) conditions of the experiments.

In general, the performance of the concentration process is characterized with the concentration factor n. For the concentration process of a solute recovery yield (*k*), the following mass balance is established:

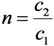


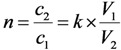
(19)
where *V*_1_ and *V*_2_ are the solution volumes before and after concentration, respectively. *c*_1_ is the solute concentration in the solution before the concentration and *c*_2_ is the solute concentration in the solution after the concentration using a given concentration process.

In individual case of ^99m^Tc concentration, *c*_1_ is the ^99m^Tc radioactivity concentration in the eluate eluted from the ^99m^Tc generator and *c*_2_ is the ^99m^Tc radioactivity concentration in the ^99m^Tc solution concentrated using a given concentration process. 

Relating the above equations, the following is derived:

*V*_2_ × *c*_2_ = *k* × *c*_1_ × *V*_1_ (20)

Except being concentrated by the evaporation of solvent or by electrolysis, all chromatographic column concentration processes are described by the following basic equations:

For a sorbent (e.g., ion-exchange resin) characterized with a volume of solid substrate used in the concentration column:

*V*_1_ = *V_m_* + *K_V_* × *V_S_* (21)

For a sorbent (e.g., alumina) characterized with a specific surface area of solid substrate used in the concentration column:

*V*_1_ = *V_m_* + *K_S_* × *S* (22)

where:

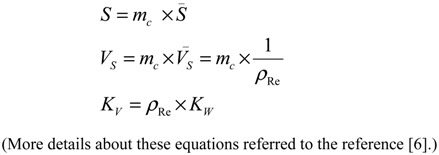



The following is received by relating Equations (21) and (22):

*K_V_* × *V_S_* = *K_S_* × *S*

*K_S_* value is calculated by putting the value of *K_V_*, *V_S_*, and *S* into this equation:

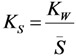
(23)
where *K_S_* (mL/m^2^), *K_V_* (mL/mL), *K_W_* (mL/g) is the area, volume, weight distribution coefficient of the solute (^99m^TcO_4_^−^) in a given sorbent-solution system, respectively;

*S* is the surface area of the sorbent (m^2^);
*V_s_* is the volume of the dry resin (mL);
*m_c_* is the weight of the dry resin/sorbent loaded in the column (g);
*S* is the specific surface area of the sorbent (m^2^/g);
*V*_s_ is the specific volume of the resin (mL/g);
*ρ*_Re_ is the weight density of the resin (g/mL).

Based on the above equations (assuming the dead volume of the concentration column *V_m_ << V*_2_), the concentration factor (*n*) is assessed for designing the concentrator column as follows: 

For the ion-exchange resin column:

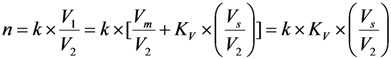
(24)

For the sorbent column:

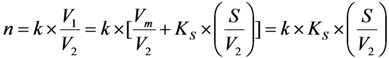
(25)

If *V*_2_ is given as a designed value, the concentration factor (*n*) only depends on the value of *k*, *K_S_* and *S* (or *K_V_* and *V_S_*).

Due to the diversity of the eluents of variable volume used for the elution of ^99m^Tc-generators, the evaluation of concentration factor of the integrated generator systems (integrated elution-concentration processes) should be harmonized using a common language for communication/justification on the elution/concentration performance of the given systems. When a non-saline solvent-eluted process is applied for the ^99m^Tc generator elution and that consecutively the eluate of this elution is concentrated using a chromatographic column concentration method, we need a tool to assess/justify the effectiveness of each elution-concentration process in comparison with others. We need then a reference to be used for the comparison. The saline-eluted process of the ^99m^Tc generator is considered as a gold standard/reference elution due to its suitability for clinical use. The reference is set up as follows:

*V_Eqv_* (equivalent volume) is the volume of non-saline eluent used for the elution of ^99m^Tc from a generator (with a non-specified activity) giving a ^99m^Tc elution yield *f_E_* which is equal to the yield achieved by an elution performed with the volume *V_S_*_1_ of saline.

*V_E_* is the volume of non-saline eluent (containing ^99m^Tc) actually passed through the concentration column of weight *m*, in which ^99m^Tc will be retained with adsorption yield (*x*) from its total amount present in the volume *V_E_*.

At the stage of the elution of the concentration column with a small volume of saline, *V_S_*_2_ is the volume of the saline used to recover the ^99m^Tc from the concentration column to achieve a concentrated ^99m^Tc solution and the elution yield of this concentration column is *y*. The yield of the overall concentration process *k* is composed of the adsorption yield *x* and recovery elution yield *y*, as follows:

*k* = *x* × *y*

The normalized concentration factor will be set up as follows:

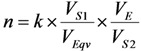
(26)

With introduction of the weight of the sorbent (*m*) used in the concentration column, the further analysis of the above equation is shown as follows:

*V* × *m* = *V_S_*_2_

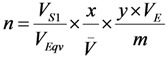
(27)
where, *V* (mL/g) is the specific elution volume of the concentration column eluted with saline to get a concentrated ^99m^Tc solution of volume *V*_2*S*_.

Equation (27) comprises four components characterizing the system involved:

The term (*V_S_*_1_/*V_Eqv_*) characterizes the relation of the saline elution *versus* alternative non-saline elution of a given generator column.

The term (1 / *V*) characterizes the saline elution of the concentrator column.

(*V_E_*/*m*) and *k* characterize the adsorption/elution capability of the sorbent for the pertechnetate ions with an alternative non-saline eluent.

The equations described above can be used for both theoretical and practical evaluations of the normalized concentration factor:

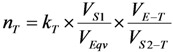
(28)

Equation (28) is used for theoretical assessment of the normalized concentration factor. The terms *k_T_* = 1; *V_E−T_* and *V_S_*_2*−T*_ are obtained from the practical determination of retention time/retention volume using an established standard chromatographic procedure performed with the same column or are calculated from the distribution coefficient *K* as described above. *K_W_* is determined as described in literature [6]. *n_T_* value is used for the evaluation of the effectiveness of the concentration system (sorbent-eluent)/method of interest, while *n_P_* value is to evaluate the performance of a practical procedure/concentrator device designed using this concentration system/method. *n_P_* value is calculated as follows:

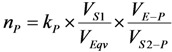
(29)
where, *V_E−P_* and *V_S_*_2*−P*_ are the volume of non-saline eluent and saline actually used in the concentration procedure/device, respectively. 

Note that the overall ^99m^Tc recovery yield of the integrated generator-concentration system will be:

*Y* = *f_E_* × *k* (30)

where, *f_E_* is the elution yield of the generator column and *k* is the purification/concentration yield.

As the outcomes of the above reported evaluation process, the designs of the radioisotope concentrator ULTRALUTE^®^ and integrated radionuclide generator systems RADIGIS-^68^Ga and RADIGIS-^99m^Tc and the performance assessment of the developed concentration processes have been successfully performed in our projects of the radionuclide generator development [3,4,5,6,7,8,9,10], which are described in the section of “Experimental Methods” Section 3.2.

### 2.3. Effective Control of Radionuclidic Purity: Relationship between Detection Limit, Required Radionuclidic Impurity Limit and Measurement Certainty for the Optimisation of Decay Time and Sample Activity Used for Post-Delivery Quality Control

The control of low activity radionuclide contaminants present in the daughter nuclide product solution is challenging both the producer and user of the radionuclide generator due to the influence of high background activity generated from the high activity of the product samples used in the measurement process, such as the measurement of ^68^Ge breakthrough in the ^68^Ga solution produced from a ^68^Ge/^68^Ga generator. Usually, there are two ways to reduce the interference of dominant activity of the product daughter nuclide for measurement of impure radionuclide contamination: (a) shielding the lower energy radiation of the product daughter radionuclide (shielding method) and (b) waiting for the decay of product daughter radionuclide (decay method). As a supplementary non-spectrometric method, the shielding method can be used when the energy of radiation emitted from the product daughter radionuclide is much lower than that of the impure nuclide, as in the case of ^99^Mo contamination in ^99m^Tc product.

The decay method is used for the determination of radionuclidic contaminants of longer half life compared with that of the daughter radionuclide product. So the determination of impure radionuclides potentially contaminated in the shorter-lived daughter nuclide products separated from the generator produced from a longer-lived parent nuclide should be performed based on the decay of the majority of product nuclide radioactivity to minimize its interference.

Particularly, the decay method is suitable for the determination of radionuclidic impurity in the positron emitting radioisotope products which is produced from proton bombardment in the cyclotron. For an economic production and/or utilisation of the daughter radionuclide product, the activity of the sample use in quality control (QC) procedure should be optimised to minimize the loss of the useful product. Different positron emitters are usually co-produced from the same targets during proton induced reaction. This fact shows that the positron emitting radioisotope products have high potential for being contaminated with positron emitting impure radionuclides which also emit the 511 keV annihilation gamma-ray, so the only way for determination of impure radionuclides is that the radioactivity of the main product must be removed to minimize its interference on the measurement of impure radionuclide activity.

Until now the decay method is set up with an estimation based on the results of the repeated tests with respect to the choice of optimal measurement conditions such as required decay time, detection capability/detection limit and sample radioactivity of the daughter nuclide product of interest. This process takes a long time and is not justified as a sound scientific argument. Besides, the lack of an identified method for the setup of radionuclidic impurity QC protocol makes user, producer and legal regulator of radiopharmaceuticals involved in a shadow of doubt. It is agreed that the capability of the method for measurement of very low activity of the above mentioned impure radionuclides present in the high activity product depends on the availability of gamma spectrometer of high sensitivity and correctly developed measurement protocol.

This paper reports on our methods developed for the measurement of radionuclidic purity based on the relationship between detection limit (*L_D_*), required radionuclidic impurity limit (*L*) and measurement reliability (*R*) with respect to optimisation of the decay time (*t*) and sample activity (*A*_0,*P*_), which is used to set up a gamma-ray spectrometric measurement protocol for a post-delivery radionuclidic purity control of the daughter radionuclide product produced from radionuclide generators.

#### 2.3.1. Impure Radionuclide Detection Limit Is Invariable with QC Sample Activity which Has Influence on the Gamma-Ray Spectrometric Measurement of Impure Radionuclide Activity due to 511 keV Photo-Peak Overlap, ^68^Ge in ^68^Ga and ^44^Ti in ^44^Sc Product as the Cases

The interference of the product QC sample activity during quantifying the impure radionuclide contamination comes from the overlap of the energy peaks of photons emitted from both impure radionuclides and product radionuclide. To make sure that this influence is minimized to an acceptable extent the certainty parameter should be introduced for further assessment. The justification “non-detectable radionuclidic impurity” should be given with the defined measurement conditions specifically designed based on the detection limit *L_D_* (as the capability of a given gamma-ray spectrometer), the required certainty degree *R*, and the required radionuclidic impurity limit *L* of a given radionuclide product.

The certainty parameter *R* (%) is defined as the ratio of the impure radionuclide activity per the sum of the product and impure radionuclides, that is:

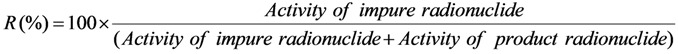


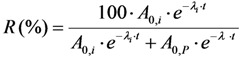
(31a)
where *A*_0,*i*_ and *A*_0,*p*_ are the activity values of the impure and product radionuclides, respectively, at the time of interest *t* = 0.

*R* is a certainty parameter, in percentage. This shows that the activity actually measured is certain to the R-percentage extent to be generated from the impure radionuclide. 

*λ_i_* and *λ* are the decay constants of the impure and product radionuclides, respectively.

With the introduction of the radionuclidic impurity limit *L* required by the national/international regulatory authority, the activity of impure radionuclide (*A*_0,*i*_) in the radioactive product is described as follows:

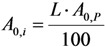


Putting the *A*_0,*i*_ value into Equation (31a), the following is obtained:

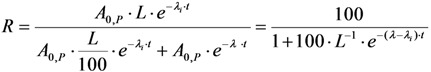
(31b)

Further re-arranging this equation, the decay time t is deduced as below:

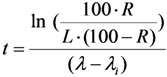
(32)

The decay time *t* in Equation (32) is the time needed to reduce the sample activity to a measure so as the result of gamma-ray spectrometric measurement of the impure radionuclide activity is confirmed with *R*% certainty. 

As shown, the decay time *t* depends only on the required impurity limit *L* and on the certainty degree *R*. It is independent on the product sample activity (*A*_0,*P*_) taken for quality control measurement. So *A*_0,*P*_ value should be pre-assessed to make sure that the following activity measurement based on gamma ray spectrometry is correctly performed regarding a reasonable spectrum acquisition life-time, counting time and background generated from Compton scattering. In principle, the assessment of value *A*_0,*P*_ is based on the mathematical equation formulated using the relationship between the detection limit *L_D_* of impure radionuclide, the decay time *t* given in Equation (32), and the required radionuclidic impurity limit *L*. 

At the measurement time point (the end of the decay time period t of the product sample for a QC procedure) the activity of impure radionuclide (*A_t_*_,*i*_) and the detection limit (*L_D_*) of this impure radionuclide determined by the given gamma-ray spectrometer are set equal, *L_D_* = *A_t_*_,*i*_. As given above, the value *A_t_*_,*i*_ is the rest of the required limit activity *A*_0,*i*_ of impure radionuclide, which has been decayed for the decay time *t*. Taking into account the above mentioned equation of the *A*_0,*i*_ *versus* *A*_0,*P*_ value, the following is formulated:

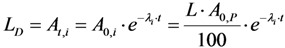
(33)

Re-arranging this equation and replacing value *t* from Equation (32), the followings are deduced:

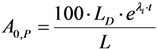


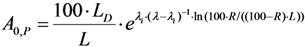
(34)

This *A*_0,*P*_ value is the minimal activity of the product QC sample at the time *t* = 0 (sampling time point) needed for the radionuclidic impurity measurement at the time *t*, which is correctly performed with an insignificant influence of the QC sample activity and with the conformation to the required certainty *R*. The experimental results of our study using the above mentioned assessment methods are reported in Section 4.3 (Results and Discussion).

#### 2.3.2. Impure Radionuclide Detection Limit Is Variable with the Activity of QC Sample which Has Radionuclides of Well-Separated Gamma-Ray Photo-Peaks, the Measurement of Different Impure Radionuclides in ^99m^Tc Product as a Case

In this case the interference of the product QC sample activity while quantifying the impure radionuclide is insignificant because no overlap of the photo-peaks has been recorded from both the impure and product radionuclides. The Compton background and coincidence gamma rays are the main interferences causing an increase in the detection limit of the impure radionuclides. To make sure that this interference is minimized to an acceptable extent the relationship between the detection limits and the QC sample activity at the measurement time should be assessed. The assessment process is performed with the help of Equation (33), described as follows (All notations used are the same as in the previous section, Section 2.3.1):

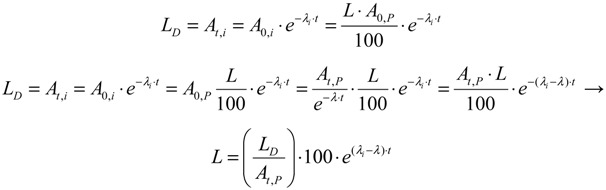


Given 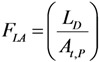, the above equation is rewritten as:

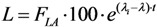
(35)

Because *λ*_i_ ˂ *λ*, it is obvious that the smaller the required limit *L* of the impure nuclide, the longer the decay time *t* of the product QC sample is required before the measurement is commenced.

Value *t* is deduced from Equation (35) as follows:

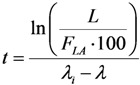
(36)


The product QC sample activity *A*_0,*P*_ at the time *t* = 0 (the delivery time point of the product) is deduced using Equation (36) and shown as follows:

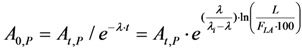
(37)

Value *A_t_*_,*P*_ is pre-determined depending on the performance of available gamma ray spectrometer regarding the counting live time at the acquisition. Value *F_LA_* is calculated from value *L* as shown in Equation (35).

*Note*: Measurement of ^99^Mo impurity in ^99m^Tc solution routinely performed by the shielding method is based on the advantage that the impure radionuclide ^99^Mo has the defined photo-peaks of higher energy than that of the product ^99m^Tc-radionuclide. Because the high activity of ^99m^Tc product QC sample will make the detector of gamma ray spectrum analyser incapable to resolve the photons emitted from the radionuclides of the measurement sample, the ^99^Mo impurity evaluation is performed by measuring the gamma rays of the ^99^Mo nuclide behind the Pb or W shielding (Housing the product sample in a Pb or W pot of relevant thickness) which is used for stopping the majority of lower energy gamma ray emitted from the ^99m^Tc product radionuclide. So the gamma ray attenuation factor *k* should be used in the assessment process:

*k =* (*I_d_* / *I*_0_) = *e^μ·d^* (38)

where *I_d_* is the intensity of gamma ray transmitted through a thickness d of shielding material. *I*_0_ is the incident intensity of gamma ray and *μ* is the absorption coefficient. 

#### 2.3.3. Impure Radionuclide Detection Limit Is Variable with the Radioactivity of QC Sample which Has Influence on the Gamma-Ray Spectrometric Measurement of the Impure Radionuclide Activity due to the Overlap of Major Photo-Peaks Emitted from both the Impure and Product Radionuclides, the Measurement of ^88^Zr/^88^Y Impurities in ^89^Zr Product as a Case

In this case the interference of the product QC sample activity while quantifying the impure radionuclide is significant due to the combined effects of the overlap of photon peaks emitted from impure and product radionuclides and the higher detection limits caused by high Compton background and coincidence gamma rays. To make sure that this interference is minimized to an acceptable extent the relationship of the detection limits *versus* the QC sample activity *A*_0,*P*_ at the measurement time should be assessed. The assessment process requires a combination of two previous assessment processes which are reported in Section 2.3.1 and Section 2.3.2. Additionally, the compromise should be applied as shown below. Putting Equation (32) equal to Equation (36) and rearranging, it is found that:

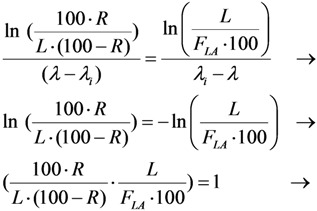


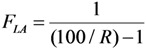
(39)

Replacing this *F_LA_* value into Equation (36), the decay time *t* is obtained in the following:

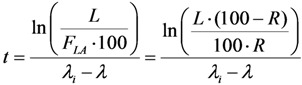
(40)

The product QC sample activity *A*_0,*P*_ at the time *t* = 0 (the delivery time point of the product) is deduced using Equation (40) and shown as follows:

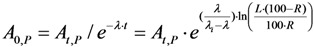
(41)

Equation (39) shows a specified value *R* because the value *F_LA_* is specified for a gamma ray spectrum analyser at a given sample activity value of the defined radionuclide product. The larger the *F_LA_* value, the higher the *R* value is achievable. This should be clarified as follows. 

Equation (39) also shows that the higher the certainty degree *R* (when approaching to 100%), the higher the value *F_LA_* will be achieved. The higher *F_LA_* value is only resulted from a newly established relation of the decayed QC sample activity *A_t_*_,*P*_ with the corresponding detection limit *L_D_* value. As a rule of thumb, *L_D_* will slower approach to a defined unchanged value corresponding to the background spectrum of a blank sample when the *A_t_*_,*P*_ value is decreased. So the use of the QC sample of smaller activity *A_t_*_,*P*_ will result in a higher *F_LA_* value.

## 3. Experimental

### 3.1. Materials and Radioactivity Measurement

Capintec dose calibrator and Ortec gamma-ray spectrometer coupled with HP Ge detector were used for radioactivity measurement. ^99^Mo/^99m^Tc and ^68^Ge/^68^Ga generators were supplied from ANSTO Health and ANSTO Life Sciences, respectively. The content of chemical elements in the daughter nuclide eluate produced from the generators were analysed by ICP-EOS method.

### 3.2. Radioisotope Generator Integrated Systems for Study on the Optimisation Assessment of the Daughter Nuclide Build-up and Production Yield

Two systems used for the purpose of optimisation assessment of the daughter nuclide build-up and production yield are RADIGIS system (Integrated Elution-Purification-Concentration System designed for a universal use purpose) and Radioisotope concentrator ULTRALUTE^®^ (developed by Medisotec and Cyclopharm Ltd., Sydney, Australia) which is in-line coupled with Gentech (ANSTO) ^99m^Tc-generator.

**Figure 7:**
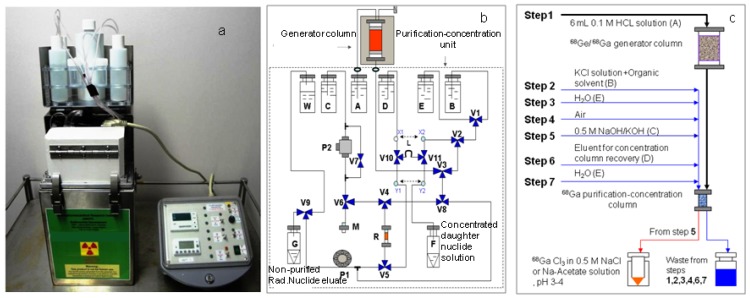
Automation process and Flow chart of the universal Radioisotope Generator. Integrated System (RADIGIS) which is programmable for different parent/daughter pairs without change in the system hardware setup: (**a**) Photo of the universal RADIGIS system developed by MEDISOTEC [8]; (**b**) Fluid flow diagram and setup of the universal RADIGIS system (P1, peristaltic pump; P2, micro displacement pump; V, solenoid valve; A–E, solutions; W, waste; L, loop for neutralization; R, specific sorbent concentration column; G, non-purified daughter nuclide eluate; F, final purified daughter nuclide solution); (**c**) Processing flowchart for the elution-purification-concentration of ^68^Ga used in RADIGIS-^68^Ga system [7,9].

Authors have successfully used the method described in Section 2.2 to design and set up the device ULTRALUTE^®^ and the RADIGIS systems for the production of daughter nuclides ^99m^Tc, ^188^Re, and ^68^Ga in a small solution volume (0.75 mL–1.0 mL) with an increased concentration of the daughter nuclide [13,14,15,16]. RADIGIS is a radioisotope separation integrated system developed for the separation, purification, and concentration of different radionuclide solutions. The fluid flow diagram and setup of a typical multipurpose RADIGIS system is shown in Figure 7b. Based on this diagram, the process of ^68^Ga elution from the generator followed by ^68^Ga eluate purification/concentration was performed using a low-cost automated benchtop radioisotope generator integrated system specially designed for elution-purification-concentration of ^68^Ga radioisotope, RADIGIS-^68^Ga [7,9]. With further development related to the sorbent materials used in the generator column for selective immobilisation of specified parent nuclide, RADIGIS systems with a programmable control system for ^99^Mo/^99m^Tc and ^188^W/^188^Re generators were also developed by MEDISOTEC [5,6,8].

*RADIGIS-^68^Ga system*: The design of the RADIGIS-^68^Ga system, which comprises a nanocrystalline sorbent generator column coupled with a cation-exchange resin purification-concentration column, was performed based on the fundamental equations as described in Section 2.2. The parameters used in the design are the followings: Distribution coefficient *K_S_* of ^68^Ga^3+^ ions (*K_S_* = 0.01 in 0.5 M KOH for the cation exchange resin AG-50W-X4 concentration column), *V*_2_ = 0.75 mL, resin weight *m* = 30 mg, *k* = 0.95. The available concentration factor for the wet elution of generator column with 0.1 M HCl solution and the elution of concentration column with 0.5 M NaOH solution is *n* = 6.1 (with *V*_1_ = 5 mL for the generator column containing 1.0 g nanocrystalline zirconia-titania composit ZT-31 sorbent). The process of ^68^Ga elution-purification-concentration is based on the much higher adsorption selectivity (or separation factor) of Ga^3+^ ions compared with other metal ions in the system of acidic alcohol aqueous solution/salt-form strong cationic exchange resin. The process of ^68^Ga purification-concentration was set up as shown in Figure 7a. ^68^Ga eluate of around 5–10 mL volume in 0.1 M HCl solution eluted from the ^68^Ge/^68^Ga generator column was purified on a small cationic exchanger column with an aqueous alcohol solution mixture of hydrochloric acid and ascorbic acids, and halide salts. An alkali solution was used for elution of ^68^Ga from the ion exchange resin column to obtain purified ^68^Ga solution, which is neutralized with acidic solution to obtain a final ^68^Ga product of pH 3–4 in 0.75 mL 0.5 M NaCl or 0.5 M sodium acetate solution. Metallic contamination of <3 nmol per mL was found in this organic-solvent-free ^68^Ga solution product of an acidity suitable for coordinative labelling of radiopharmaceuticals.

*RADIGIS-^99m^Tc system*: The design of RADIGIS-^99m^Tc system, which comprises the molybdate gel or PZC/PTC nanocrystalline sorbent generator columns coupled with a alumina concentrator column, was based on the following parameters (which are calculated using the equations mentioned in Section 2.2): *K_S_* = 2.0, *V*_2_ = 5.0 mL, *k* = 0.95. The available concentration factor of the bolus elution with *V*_1_ = 460 mL for a generator column of 375–380 g TiMo (or PZC/PTC) gel) is *n* = 55.7. For the elution-by-elution operation program (elution with *V*_1_ = 65 mL for each elution from a generator column of 53 g sorbent), a concentration factor *n* = 11.2 was achievable.

**Figure 8:**
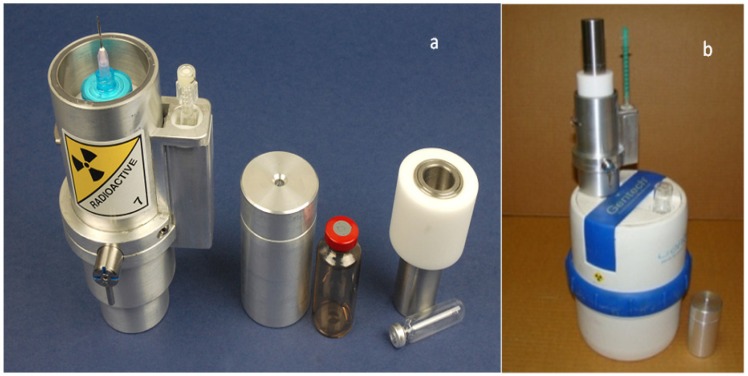
Radioisotope concentrator device ULTRALUTE^®^ [4] with its accessories (**a**) and in-line attachment of ULTRALUTE^®^ to Gentech ^99m^Tc-generator to perform an integrated elution-purification-concentration process (**b**).

*Disposable cartridge-based radioisotope concentrator ULTRALUTE^®^*: The design of the radioisotope concentrator ULTRALUTE^®^ is also performed with the formulations mentioned in Section 2.2. ULTRALUTE^®^ concentrator produced by Cyclopharm Ltd. using a concentration-column of more effective new sorbent is shown in Figure 8.

The ^99m^Tc eluate was concentrated more than 10-fold with a ^99m^Tc recovery yield of >85% using ULTRALUTE^®^ concentrator device. The increase in ^99m^Tc concentration in the eluate enhances the utilisation of technetium in Technegas generator-based lung perfusion (100–250 mCi/mL) and other SPECT (20–30 mCi/mL) imaging studies. five or 10 repeated elutions were successfully performed with each cartridge coupled to the 10 mL or 5 mL saline solution- eluted generators, respectively. So, each cartridge can be effectively used for one week in the hospital environment for radiopharmaceutical formulation. This fact also shows that when a bolus ^99m^Tc-solution is needed to be concentrated, the concentration factor *n* = 50 can be achieved. The useful lifetime of ^99m^Tc generator was significantly extended from 10 to 20 days for the generators of 300–3000 mCi activity, respectively. This means that about 20% of the generator activity is saved by extending the life time of the generator. The use of ULTRALUTE^®^ and RADIGIS systems in combination with the methods of build-up time optimisation and early elution schedule described in Section 2.1.1, Section 2.1.2 and Section 2.1.3 for the optimisation of radionuclide generator utilisation is reported in Section 4.1.2 and Section 4.2.

### 3.3. Determination of Gamma-Ray Spectrometric Detection Limit (L_D_) for the Certainty Assessment of Effective Radionuclidic Purity Test

The method of determination of counting decision limits in the gamma ray spectrum analysis (critical limit *L_C_*, upper limit *L_U_* and detection limit *L_D_*) described in the references [17,18] was applied. It is worth mentioning that the detection limit gives an answer to a priori question, whereas critical and upper limit are both posterior estimates based upon actual measured counts. As justified from definition of detection limit the range of *L_D_* > *L_U_* > *L_C_* can be stated. Although *L_U_* is the most likely to be identified with a minimum detectable activity (MDA) and that basing on expected counts it tells the fact that we might detect the activity, in this work *L_D_* is a preferable choice to be discussed for the reason of pre-evaluation of the radionuclidic impurity analysis performance for an individual radioactive product, which is ^68^Ga solution as an example. 

The decision limits (*L_D_*, *L_U_*, *L_C_*) were calculated using the following equations and spectrum counts. The calculation was made for the photo peak area of gamma rays with emission yield *Y* (*γ* rays/one decay) and counting efficiency *ε* and for 95% confidence. 

Critical limit,*L_C_* (counts) = 1.645 × [*B* (1 + *n*/2*m*)]^1/2^
*L_C_* (cps) = (*L_C_*/*t*); *L_C_* (Ci) = [*C* × *L_C_* (cps)]Upper limit,*L_U_* (counts) = *A* + 1.645 × [*A* + *B* (1 + *n*/2*m*)]^1/2^
*L_U_* (cps) = (*L_U_*/*t*); *L_U_* (Ci) = [*C* × *L_U_* (cps)]Detection limit,*L_D_* (counts) = 2.71 + 3.29 × [*B* (1 + *n*/2*m*)]^1/2^
*L_D_* (cps) = (*L_D_*/*t*); *L_D_* (Ci) = [*C* × *L_D_* (cps)]

Conversion factor *C*: *C* = [1/(*Y* × *ε* × 37 × 10^9^)]; Counting time *t* is in seconds.

Peak region:

- Channel numbers of the peak region: *n*
- Gross counts in the peak region of n channels: *G*

Background region:

- Channel number on the right side of peak region: *m*
- Counts belongs to m channels: *s*_1_
- Channel number on the left side of peak region: *m*
- Counts belongs to m channels: *s*_2_

Sum of background region counts, *S* = (*s*_1_ + *s*_2_*)*

Background correction, *B* = (*n* × *S*)/(2 × *m*)

Net peak area (counts), *A* = *G* − B 

The detection limit values were evaluated with ORTEC gamma-ray spectrometer coupled with HP Ge detector. This instrument was calibrated using ^152^Eu standard source. The obtained evaluation results reported in Section “Results and Discussion” are used for the optimisation of decay time and radioactivity of the QC sample used for post-delivery quality control process (Section 4.3). 

## 4. Results and Discussion

### 4.1. Optimisation for Improvement of Production Yield and Specific Radioactivity

#### 4.1.1. Daughter Nuclide Build-up Optimisation and New Characteristic Parameter of Formation-Decay Kinetics of Parent/Daughter Nuclide System (Referred to Section 2.1.1 and Section 2.1.2)

**Figure 9:**
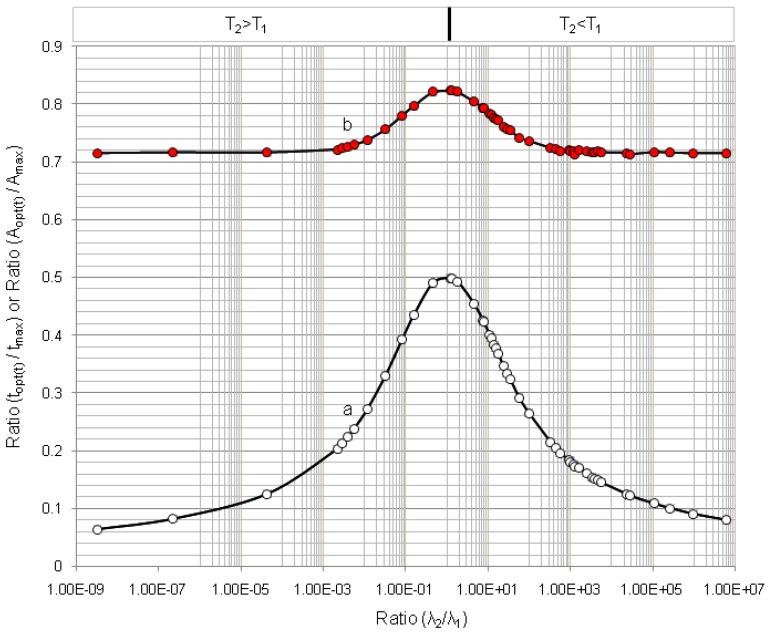
Optimal build-up time value *t_opt_*_(*t*)_ and radioactivity value *A*_2,*opt*(*t*)_ of daughter nuclides compared with values *t*_max_ and *A*_2,max_, respectively, in function of decay constant ratio (*λ*_2_/*λ*_1_): (a) Ratio (*t_opt_*_(*t*)_/*t*_max_); (b) Ratio (*A*_2,*opt*(*t*)_/*A*_2,max_); *T*_1_ and *T*_2_ are the half-life times of the parent and daughter nuclides, respectively.

**Figure 10:**
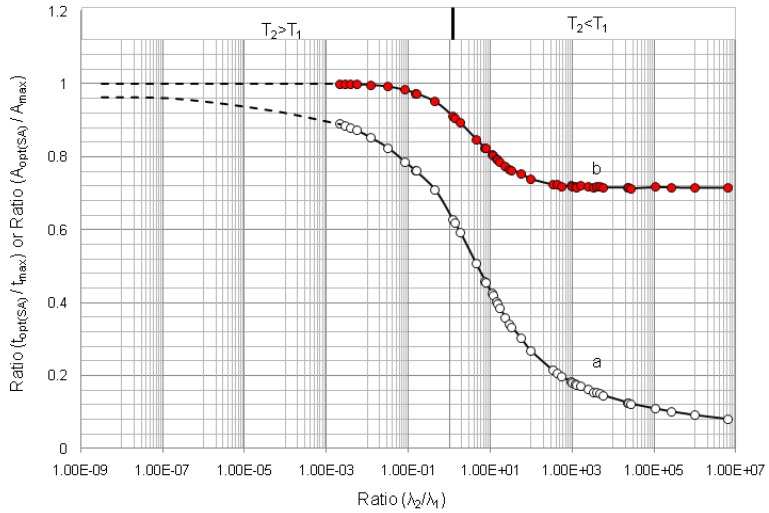
Optimal build-up time *t_opt_*_(*SA*)_ and radioactivity *A*_2,*opt*(*SA*)_ of daughter nuclides compared with *t*_max_ and *A*_2,max_ values, respectively, in function of decay constant ratio (*λ*_2_/*λ*_1_): (a) Ratio (*t_opt_*_(*SA*)_/*t*_max_); (b) Ratio (*A*_2,*opt*(*SA*)_/*A*_2,max_); *T*_1_ and *T*_2_ are the half-life times of the parent and daughter nuclides, respectively.

**Figure 11:**
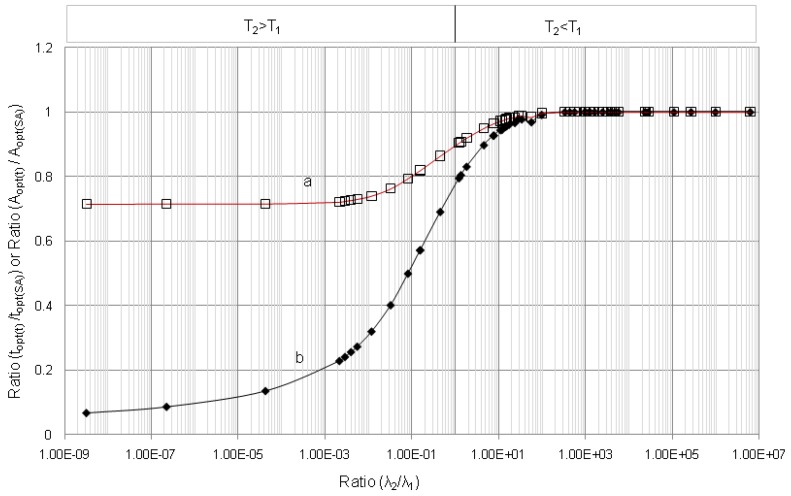
Ratio of optimal build-up times (*t_opt_*_(*t*)_/*t_opt_*_(*SA*)_) and Ratio of optimal build-up radioactivity (*A*_2,*opt*(*t*)_/*A*_2,*opt*(*SA*)_) of daughter nuclides in function of decay constant ratio (*λ*_2_/*λ*_1_): (a) Ratio (*t_opt_*_(*t*)_/*t_opt_*_(*SA*)_); (b) Ratio (*A*_2,*opt*(*t*)_/*A*_2,*opt*(*SA*)_); *T*_1_ and *T*_2_ are the half-life times of the parent and daughter nuclides, respectively.

The results of optimisation assessment for 50 parent/daughter nuclide pairs of different half-lives are reported in Table 1 and in Figure 9, Figure 10 and Figure 11. The diagrams of the build-up time ratio (*t_opt_*_(*t*)_/*t*_max_) and daughter nuclide activity ratio (*A*_2,*opt*(*t*)_/*A*_2,max_) plotted against decay constant ratio (*λ*_2_/*λ*_1_), which are based on the results of the optimisation of the daughter nuclide activity build-up *versus* build-up time reported in Table 1, are shown in Figure 9. The plots show defined characteristics of daughter nuclide build-up kinetics in relation with the decay constant ratio (*λ*_2_/*λ*_1_) of parent/daughter nuclide systems. The diagram in Figure 9 reveals a maximum value (*t_opt_*_(*t*)_/*t*_max_) = 0.5 at the ratio value (*λ*_2_/*λ*_1_) = 1 and a variation in the (*t_opt_*_(*t*)_/*t*_max_) values from 0.05 to 0.5 for the whole range of different parent/daughter nuclide pairs. Accordingly, a maximum value (*A*_2,*opt*(*t*)_/*A*_2,max_) = 0.823 at the ratio value (*λ*_2_/*λ*_1_) = 1 and a variation in the (*A*_2,*opt*(*t*)_/*A*_2,max_) values from 0.714 to 0.823 for the whole range of different parent/daughter nuclide pairs are presented. Both diagrams in Figure 9 show a beautiful symmetric shape irrespective of the daughter nuclide living longer or shorter than its parent. 

This characteristics means that the value (*t_opt_*_(*t*)_/*t*_max_) and accordingly the value (*A*_2,*opt*(*t*)_/*A*_2,max_) only depend on the decay constant ratio (*λ*_2_/*λ*_1_) of parent/daughter nuclide systems, but not on any other specified parameter of the parent or its daughter nuclide. This statement is clearly justified by Equation (11) and thus *the value t_opt(t)_ is proved as a characteristic physical parameter of the formation-decay process kinetics of given parent/daughter nuclide pair system*.

**Table 1:** Optimal build-up time and radioactivity of daughter nuclide in different radionuclide generator systems: *t*_max_, *t_opt_*_(*t*)_, and *t_opt_*_(*SA*)_ values are calculated using Equations (2), (11) and (14), respectively. *A*_2,max_, *A*_2,*opt*(*t*)_, and *A*_2,*opt*(*SA*)_ are the daughter nuclide activities at the build-up time *t*_max_, *t_opt_*_(*t*)_, and *t_opt_*_(*SA*)_, respectively, which are calculated using Equation (1) and relevant build-up time values. The half-life time data are from References [19,20].

Radioisotope Parent *R*_1_-Daughter *R*_2_	Half life		*λ*_2_*/λ*_1_	*t_max_*	*t_opt_*_(*t*)_	(*t_opt_*_(*t*)_/*t_max_*)	(*A*_2_,*_opt_*_(*t*)_/*A*_2_,_max_)	*t_opt_*_(*SA*)_	(*t_opt_*_(*SA*)_*/t_max_*)	(*A*_2_,*_opt_*_(*SA*)/_*A*_2_,_max_)
	Parent Nuclide *T*_1_	Daughter Nuclide *T*_2_								
^99m^Tc–^99^Tc	6.02 h	214000 y	3.21 × 10^−9^	170.09	10.91	0.064	0.714	-	-	-
^93m^Mo–^93^Mo	6.9 h	3500 y	2.25 × 10^−7^	152.14	12.51	0.082	0.715	-	-	-
^83m^Sr–^83^Sr	5.0 s	1.35 d	4.27 × 10^−5^	0.0201	0.0025	0.125	0.715	-	-	-
^131^Te–^131^I	25 min	192.96 h	2.16 × 10^−3^	3.6984	0.75	0.203	0.721	3.29	0.889	0.999
^82m^Br–^82^Br	6.13 min	35.3 h	2.90 × 10^−3^	0.8641	0.184	0.219	0.723	-	-	-
^67^Ge–^67^Ga	18.7 min	78.26 h	3.98 × 10^−3^	2.4955	0.56	0.224	0.725	2.19	0.877	0.998
^57^Ni–^57^Co	36.16 h	270.9 d	5.56 × 10^−3^	272.10	64.73	0.238	0.729	237.5	0.872	0.998
^125^Xe–^125^I	17 h	59.89 d	1.18 × 10^−2^	110.13	30	0.272	0.737	94	0.853	0.996
^149^Nd–^149^Pm	1.73 h	53.08 h	3.26 × 10^−2^	8.8358	2.92	0.330	0.756	7.28	0.823	0.992
^95^Ru–^95^Tc	1.63 h	20 h	8.15 × 10^−2^	6.4218	2.52	0.392	0.780	5.05	0.786	0.982
^131m^Te–^131^I	30 h	8.04 d	1.55 × 10^−1^	95.425	41.52	0.435	0.797	72.65	0.761	0.973
^123^Xe–^123^I	2.08 h	13.3 h	1.56 × 10^−1^	6.6033	2.87	0.435	0.797	5.02	0.760	0.973
^52^Ti–^52^V	1.7 min	3.76 min	4.52 × 10^−1^	0.0592	0.029	0.489	0.821	0.042	0.709	0.950
^131^Ba–^131^Cs	11.8 d	9.69 d	1.22	369.75	184.5	0.499	0.823	232.1	0.627	0.911
^47^Ca–^47^Sc	4.536 d	3.351 d	1.35	134.63	67	0.498	0.823	83.25	0.618	0.906
^95^Zr–^95^Nb	64.05 d	34.98 d	1.83	1613.3	795.1	0.493	0.821	957.2	0.593	0.893
^38^S–^38^Cl	169.7 min	37.24 min	4.56	1.7401	0.79	0.454	0.804	0.88	0.505	0.847
^14^°Ba–^14^°La	12.74 d	40.272 h	7.59	135.65	57.61	0.425	0.793	62.16	0.458	0.823
^72^Se–^72^As	8.4 d	26 h	7.76	88.231	37.3	0.423	0.792	40.2	0.455	0.821
^99^Mo–^99m^Tc	66.02 h	6.007 h	11.00	22.849	9.145	0.400	0.783	9.685	0.423	0.805
^115^Cd–^115m^In	53.46 h	4.486 h	11.90	17.460	6.9	0.395	0.781	7.3	0.418	0.803
^97^Zr–^97^Nb	16.9 h	72.1 min	14.10	4.9339	1.89	0.383	0.776	1.98	0.401	0.794
^8^°^m^Br–^8^°Br	4.42 h	17.4 min	15.20	1.2196	0.461	0.378	0.774	-	-	-
^125^Sb–^125m^Te	2.77 y	58 d	17.40	6090.6	2245	0.368	0.771	2335	0.383	0.786
^52^Fe–^52m^Mn	8.3 h	21.1 min	23.60	1.6755	0.58	0.346	0.761	0.6	0.358	0.774
^87^Y–^87m^Sr	80.3 h	2.8 h	28.60	14.069	4.7	0.334	0.757	4.8	0.341	0.765
^132^Te–^132^I	78.2 h	2.3 h	34.10	12.063	3.91	0.324	0.754	4	0.331	0.762
^62^Zn–^62^Cu	9.2 h	9.7 min	56.90	0.9595	0.28	0.292	0.741	0.289	0.301	0.753
^188^W–^188^Re	69.4 d	16.98 h	98.20	113.49	30.1	0.265	0.736	30.35	0.267	0.739
^122^Xe–^122^I	20.1 h	3.6 min	3.35 × 10^2^	0.5049	0.108	0.214	0.723	0.108	0.213	0.723
^1^°^3^Pd–^1^°^3m^Rh	16.96 d	56.12 min	4.37 × 10^2^	8.2235	1.69	0.205	0.722	1.69	0.205	0.722
^28^Mg–^28^Al	20.91 h	2.24 min	5.60 × 10^2^	0.3415	0.067	0.196	0.718	0.067	0.196	0.718
^128^Ba–^128^Cs	2.4 d	3.6 min	9.58 × 10^2^	0.5950	0.109	0.183	0.720	0.109	0.183	0.720
^1^°^3^Ru–^1^°^3m^Rh	39.35 d	56.12 min	1.01 × 10^3^	9.3456	1.692	0.181	0.718	1.692	0.181	0.718
^1^°^9^Pd–^1^°^9m^Ag	13.43 h	39.8 s	1.21 × 10^3^	0.1133	0.02	0.176	0.718	0.02	0.176	0.718
^81^Rb–^81m^Kr	4.6 h	13.1 s	1.27 × 10^3^	0.0375	0.0065	0.173	0.713	0.0065	0.173	0.713
^113^Sn–^113m^In	115.1 d	1.7 h	1.62 × 10^3^	18.146	3.1	0.171	0.720	3.1	0.170	0.720
^118^Te–^118^Sb	6 d	3.5 min	2.47 × 10^3^	0.6577	0.106	0.161	0.718	0.106	0.161	0.718
^178^W–^178^Ta	21.5 d	9.3 min	3.33 × 10^3^	1.8148	0.28	0.155	0.715	0.28	0.154	0.715
^9^°Sr–^9^°Y	28.82 y	64 h	3.95 × 10^3^	763.72	116	0.152	0.717	116	0.151	0.717
^195m^Hg–^195m^Au	40 h	30.6 s	4.71 × 10^3^	0.1037	0.0155	0.149	0.718	0.0155	0.149	0.718
^68^Ge–^68^Ga	271 d	68.3 min	5.71 × 10^3^	14.211	2.063	0.145	0.716	2.063	0.145	0.716
^42^Ar–^42^K	32.9 y	12.36 h	2.34 × 10^4^	179.43	22.4	0.125	0.715	22.4	0.124	0.715
^144^Ce–^144^Pr	284.9 d	17.28 min	2.37 × 10^4^	4.1869	0.522	0.125	0.715	0.522	0.124	0.715
^82^Sr–^82^Rb	25 d	1.3 min	2.77 × 10^4^	0.3198	0.039	0.122	0.712	0.039	0.121	0.712
^44^Ti–^44^Sc	48.2 y	3.9 h	1.09 × 10^5^	65.068	7.07	0.109	0.716	7.07	0.108	0.716
^191^Os–^191m^Ir	15.4 d	4.96 s	2.68 × 10^5^	0.0248	0.0025	0.101	0.715	0.0025	0.100	0.715
^1^°^9^Cd–^1^°^9m^Ag	453 d	39.8 s	9.82 × 10^5^	0.2201	0.02	0.091	0.714	0.02	0.090	0.714
^137^Cs–^137m^Ba	30.14 y	2.552 min	6.20 × 10^6^	0.9599	0.077	0.080	0.714	0.077	0.080	0.714

The rule controlled in the relationship between the (*t_opt_*_(*t*)_/*t*_max_) and (*λ*_2_/*λ*_1_) values described in Equation (11) is independent on the fact that the daughter nuclide lives longer or shorter than its parent and that how long they live. In other words, this rule is the following: 

The shorter or longer the daughter nuclide lives compared with the lifetime of its parent, the shorter is the optimal build-up time *t_opt_*_(*t*)_ of daughter nuclide compared with its maximal build-up time *t*_max_. The value of ratio (*t_opt_*_(*t*)_/*t*_max_) is 0.5 for any parent/daughter nuclide pair of equal lifetime. The value of ratio (*t_opt_*_(*t*)_/*t*_max_) is 0.05 for any parent/daughter nuclide pair of a lifetime difference of >3 × 10^8^ times.

Accordingly, the shorter or longer the daughter nuclide lives compared with the lifetime of its parent, the smaller is the build-up activity achieved at the time *t_opt_*_(*t*)_ of daughter nuclide compared with its maximal build-up activity at the time *t*_max_ .The value of ratio (*A*_2,*opt*(*t*)_/*A*_2,max_) is 0.823 for any parent/daughter nuclide pair of equal lifetime. The value of ratio (*A*_2,*opt*(*t*)_/*A*_2,max_) is 0.70 for any parent/daughter nuclide pair of a lifetime difference of >3 × 10^8^ times.

It is found that at the optimal build-up time *t_opt_*_(*t*)_ the daughter nuclide activity varies in the range from 71.4% to 82.23% of the maximal build-up activity at the maximal time *t*_max_, whereas the optimal build-up time *t_opt_*_(*t*)_ changes only from 5% to 50%. This fact leads to a useful application in the cost-effective operation of radionuclide generators that all generator elutions could be performed at the optimal build-up time *t_opt_*_(*t*)_ instead of being performed at the maximal time *t*_max_. The elutions performed at optimal build-up time *t_opt_*_(*t*)_ result in a significant saving in the standby time of the generator, thus economic use of the generator. The following examples will clarify the effectiveness of the elution plan performed at the optimal build-up time *t_opt_*_(*t*)_.

**Figure 12:**
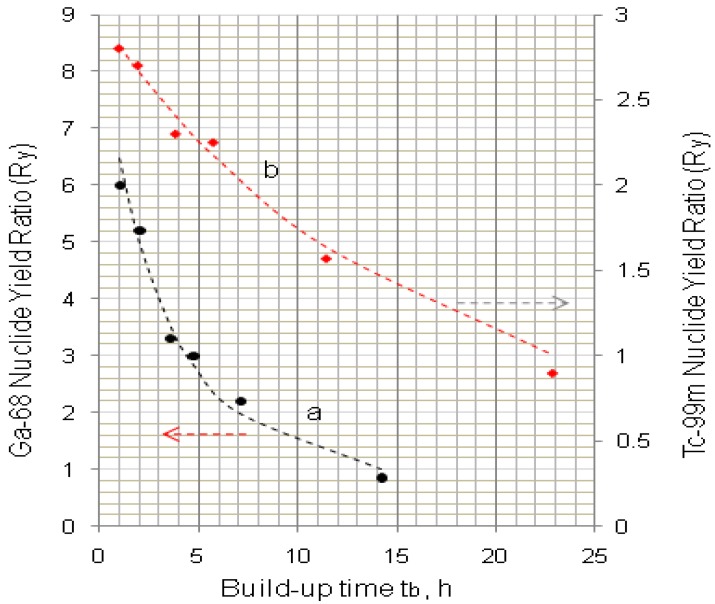
Effectiveness of daughter nuclide activity utilisation of generators eluted with an early elution schedule compared with that normally eluted at the maximal time of daughter nuclide build-up (Data mark-points are experimental and dashed lines are theoretical calculation results): (**a**) ^99m^Tc from ^99^Mo/^99m^Tc generator; (**b**) ^68^Ga from ^68^Ge/^68^Ga generator.

First example for the case of the parent and daughter nuclides of moderate difference in their half lives: The maximal build-up time of ^99m^Tc nuclide in ^99^Mo^/99m^Tc generator system is *t*_max_ = 22.86 h. The ^99m^Tc radioactivity build-up for the elution performed at optimal build-up time *t_opt_*_(*t*)_ = 9.145 h is 78.3% of the maximal build-up at the time *t*_max_ = 22.86 h. This fact dictates that the elution performed at optimal build-up time *t_opt_*_(*t*)_ saves 13.715 h or 60% of standby time of the generator whereas the optimal ^99m^Tc-activity build-up loses only 21.7% compared with the elution performed at the time *t*_max_. Despite the 21.7% loss in each elution performed at the optimal time *t_opt_*_(*t*)_, the total yields of consecutive elutions performed at optimal 9.145-h build-up time will be 1.75 times higher than the yield of one elution performed at the time *t*_max_ as evaluated by the method of early elution schedule described in Section 2.1.3 (Results shown in Figure 12).

Second example for the case of the parent and daughter nuclides of big difference in their half life: The maximal build-up time of ^68^Ga nuclide in ^68^Ge/^68^Ga generator system is *t*_max_ = 14.1 h. The ^68^Ga radioactivity build-up for the elution performed at optimal build-up time *t_opt_*_(*t*)_ = 2.063 h is 71.6% of maximal build-up at the time *t*_max_ = 14.1 h. This fact dictates that the elution performed at optimal build-up time *t_opt_*_(*t*)_ saves 12.037 h or 85.5% of standby time of the generator, whereas the optimal ^68^Ga–build-up activity loses only 28.4% compared with the elution performed at the time *t*_max_. Despite the 28.4% loss in each elution performed at the optimal time *t_opt_*_(*t*)_, the total yields of consecutive elutions performed at 2.063-h optimal build-up time will be 5.0 times higher than the yield of one elution performed at the time *t*_max_ as evaluated by the method of early elution schedule described in Section 2.1.3 (Results shown in Figure 12). In conclusion, it is stated that the optimal build-up time *t_opt_*_(*t*)_ defined by the mathematical equation:

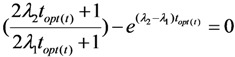

is the second characteristic parameter of the kinetics of formation-decay process of the parent/daughter nuclide system, which has a defined physical meaning and can be effectively used in the optimal management of practical radionuclide generator operation . This parameter and the first one (the maximal build-up time *t*_max_ = [ln(*λ*_2_ / *λ*_1_)] / (*λ*_2_ − *λ*_1_)) are equally significant to better understand the kinetics of the formation-decay process of parent/daughter nuclide systems and to be used in the practice of radionuclide generator production and application as well.

The diagrams of the build-up time ratio (*t_opt_*_(*SA*)_/*t*_max_) and build-up radioactivity ratio (*A*_2,*opt*(*SA*)_/*A*_2,max_) plotted against decay constant ratio (*λ*_2_/*λ*_1_), which are based on the results of the optimisation of daughter nuclide activity build-up *versus* specific activity (or *versus* total build-up daughter nuclide atom numbers per unit of daughter nuclide build-up activity) reported in Table 1, are shown in Figure 10. The plots show defined characteristics of daughter nuclide build-up kinetics in relation with the decay constant ratio (*λ*_2_/*λ*_1_) of parent/daughter nuclide systems. The diagrams reveals the upper limits of ratio (*t_opt_*_(*SA*)_/*t*_max_) ≈ 0.96 and ratio (*A*_2,*opt*(*SA*)_/*A*_2,max_) ≈ 1 at the ratio value (*λ*_2_/*λ*_1_) < 10^−9^ and the lower limits of ratio (*t_opt_*_(*SA*)_/*t*_max_) ≈ 0.05 and ratio (*A*_2,*opt*(*SA*)_/*A*_2,max_) ≈ 0.714 at the ratio value (*λ*_2_/*λ*_1_) > 10^7^. A variation in the (*t_opt_*_(*SA*)_/*t*_max_) values from 0.05 to 0.96 for the whole range of different parent/daughter nuclide pairs and the (*A*_2,*opt*(*SA*)_/*A*_2,max_) values from 0.714 to 1.0 have been noted accordingly. Both diagrams show no maximum and they are inflected at the (*λ*_2_/*λ*_1_) ratio value between (*λ*_2_/*λ*_1_) = 1 and (*λ*_2_/*λ*_1_) = 10. The values (*t_opt_*_(*SA*)_/*t*_max_) = 0.627 and (*A*_2,*opt*(*SA*)_/*A*_2,max_) = 0.911 are found at the ratio value (*λ*_2_/*λ*_1_) = 1. The rule controlled in the relationship between the (*t_opt_*_(*SA*)_/*t*_max_) values (and accordingly (*A*_2,*opt*(*SA*)_/*A*_2,max_) values) and (*λ*_2_/*λ*_1_) values described in Equation (14) is the following: 

The shorter the daughter nuclide lives compared with the life time of its parent, the smaller is the optimal build-up time *t_opt_*_(*SA*)_ of daughter nuclide compared with its maximal build-up time *t*_max_. Accordingly, the shorter the daughter nuclide lives compared with the life time of its parent, the smaller is the build-up activity achieved at the optimal time *t_opt_*_(*SA*)_ of daughter nuclide compared with its maximal build-up activity at the time *t*_max_. The value of ratio (*A*_2,*opt*(*SA*)_/*A*_2,max_) is achievable in the range 0.714–1.0 for all parent/daughter nuclide pairs.

These results mean that the value (*t_opt_*_(*SA*)_/*t*_max_) and accordingly (*A*_2,*opt*(*SA*)_/*A*_2,max_) only depend on the decay constant ratio (*λ*_2_/*λ*_1_) of parent/daughter nuclide systems, but not on any other specified parameter of parent or its daughter nuclides, with an assumption of using the specific activity formulation which is based on the equality between the total atom numbers of all involved daughter nuclides and the atom numbers of decayed parent nuclides *N* = *N*_1,0_ × (1 − *e*^−*λ*_1_*·t*^) as described in Section 2.1 (the isomer transformations of parent nuclides to form daughter nuclides are excluded). Obviously, this assumption makes the optimal build-up time *t_opt_*_(*SA*)_ become a non-characteristic parameter in a general meaning. However, it still plays an important role in the practical application in the production and use of radionuclide generators, because it is specifically characterised for a specified nuclear transformation process which generates a daughter nuclide of total atom numbers equal to the numbers of decayed parent nuclides as mentioned above. This justification is clarified by a coordinative discussion on the combination of *t_opt_*_(*SA*)_ with the characteristic parameter *t_opt_*_(*t*)_ mentioned previously in this section.

As shown in Figure 11, the difference between *t_opt_*_(*t*)_ and *t_opt_*_(*SA*)_ values is small for the range of half-lives *T*_1_ > *T*_2_ (or *λ*_2_ > *λ*_1_). So the use of *t_opt_*_(*t*)_ and *t_opt_*_(*SA*)_ values in the optimal daughter nuclide build-up management of practical generator production and utilisation is harmonised in term of effective and optimal generator use for an improved quality (*i.e.*, specific activity) of daughter nuclide solution.

#### 4.1.2. Early Elution Schedule for Improvement of Daughter Nuclide Production Yield and Specific Radioactivity, Elution of ^99^Mo/^99m^Tc and ^68^Ge/^68^Ga Generator-Concentrator Systems as the Cases

The early elution schedule method described in Section 2.1.3 is used to evaluate the total yield of daughter nuclides produced from the generator by performing multiple elutions/separations. The partial build-up radioactivities of daughter nuclides at given standby times are eluted and the sum of all radioactivities obtained is compared with the radioactivity obtained in the elution performed at maximal build-up time (*t*_max_). The calculation (using Equations (15)–(17), Section 2.1.3) and experimental results of *Ry* yield ratio assessment are reported in two typical examples below.

*Example 1. ^99^Mo/^99m^Tc generator-concentrator system*: The maximal build-up time of ^99m^Tc daughter nuclide in ^99^Mo^/99m^Tc generator system is *t*_max_ = 22.86 h. ^99m^Tc build-up radioactivities achieved in the consecutive elutions performed at optimal ^99m^Tc-radioactivity build-ups 78.3% (at *t_opt_*_(*t*)_ = 9.145 h) and 80.5% (at *t_opt_*_(*SA*)_ = 9.685 h) are shown in Table 1 and Figure 1. Despite the optimal build-up times of ^99m^Tc daughter nuclide in ^99^Mo^/99m^Tc generator system between 40.0% and 42.3% compared with maximal build-up time as evaluated in the above mentioned optimisation assessment, more effective utilisation of hot ^99m^Tc atoms may be found if only a partial build-up of ^99m^Tc in the generator is allowed to occur at a build-up time shorter than optimal build-up time (<9.145 h) before milking the ^99m^Tc from the generator column and repeating this partial elution (early elution) several times. As an example, the ^99m^Tc radioactivity build-up 61.5% at build-up time *t_b_* = 6 h (compared with a relevant maximal ^99m^Tc activity in the generator) for a consecutive early elution schedule performed at 6-h build-up time are described in Figure 5. The results of *R_y_* parameter evaluation based on Equations (15)–(17) in Section 2.1.3 are shown in Figure 12. As shown, the ^99m^Tc yield of the generator eluted with a early elution schedule of build-up/standby time <6 h increases by a factor >2. Another advantage of consecutive early elutions performed at ^99m^Tc activity build-up 61.5% (compared with maximal ^99m^Tc build-up value) is the improvement in quality of ^99m^Tc solution in terms of higher ^99m^Tc specific activity, 200 Ci/μmol compared with 150 Ci/μmol obtained in an elution at *t*_max_ = 22.86 h.

*Example 2. ^68^Ge/^68^Ga generator-concentrator system*: The maximal build-up time of ^68^Ga daughter nuclide in ^68^Ge/^68^Ga generator system is *t*_max_ = 14.1 h. ^68^Ga build-up radioactivity achieved in the consecutive elutions performed at optimal ^68^Ga-radioactivity build-up 71.6% (at *t_opt_*_(*t*)_ = *t_opt_*_(*SA*)_ = 2.063 h) are shown in Table 1 and Figure 2. Despite the optimal build-up times of ^68^Ga daughter nuclide in ^68^Ge/^68^Ga generator system at 14.5% of maximal build-up time as evaluated in the above mentioned optimisation assessment, more effective utilisation of hot ^68^Ga atoms may be found if only partial build-up of ^68^Ga in the generator is allowed to occur at a build-up time shorter than optimal build-up time (<2.063 h) before milking the ^68^Ga from the generator column and repeating this partial elution (early elution) several times. As an example, the ^68^Ga radioactivity build-up 50.0% at build-up time *t_b_* = 1.128 h (compared with a relevant maximal ^68^Ga activity in the generator) for a consecutive early elution schedule performed at 1.128-h build-up time are evaluated. The results of *R_y_* parameter evaluation based on Equations (15)–(17) in Section 2.1.3 are shown in Figure 12. This elution schedule can result in a much higher overall ^68^Ga radioactivity yield and satisfactory ^68^Ga/^68^Zn ratio. The total useful ^68^Ga radioactivity gathered in 12 consecutive elutions (overall time period around 14 h) is six times higher than that eluted once at time *t*_max_ (Figure 12). Thus this elution plan offers cost-effective utilisation of the generator system. Another advantage of consecutive elution performed at 50% ^68^Ga build-up is the improvement in the quality of ^68^Ga solution in terms of reduction of Zn content in the eluate. An amount of 6.97 × 10^−3^ nmol Zn-68 was evaluated at the 50% ^68^Ga build-up compared with the value of 0.2 nanomoles at the maximum ^68^Ga build-up time *t*_max_ = 14.1 h for the 100 mCi ^68^Ge/^68^Ga generator system. With this elution plan, a ^68^Ga/^68^Zn molar ratio of approximately 3.0 in the eluate is achievable compared with the value of 0.1 at the time t_100%__build-up_. Moreover, this 1.128 h-elution plan ( with 50% ^68^Ga build-up) conforms with the time required for labelling peptide radiopharmaceutical plus consequently performing PET imaging (around 1.0 h in total) before starting a subsequent ^68^Ga generator elution. This statement is based on a unique dose of ^68^Ga-peptide injection prepared from the elution of 18 mCi activity ^68^Ga generator as a whole.

### 4.2. Use of Daughter Nuclide Concentration Process in Combination with Methods of Build-up Time Optimisation and Early Elution Schedule for Increasing the Effectiveness of Generator Utilisation, ^99^Mo/^99m^Tc and ^68^Ge/^68^Ga Generator-Concentrator Systems as the Cases (Referred to Section 2.1.1, Section 2.1.2 and Section 2.1.3 and Section 2.2 and Section 3.2)

As discussed above, the build-up time optimisation is to shorten the build-up time of daughter nuclide ( or the standby time of the generator) while maintaining the activity build-up and specific activity of daughter nuclide at a reasonably high value achievable in an optimal specified elution/separation process compared with the elution/separation performed at maximal build-up time. By another approach, the early elution schedule method deals with increasing the total elution/separation yield using a generator operation program of multiple elutions/separations which can be performed at several build-up times shorter than maximal build-up time of daughter nuclide.

Both methods are definitely useful for the operation of the generators which have a reasonably high radioactivity and are capable for the production of daughter nuclide solution with a purposely (medically) useful radioactivity concentration achievable in each elution/separation procedure. However, the multiple early (partial) elutions/separations performed at the build-up time shorter than the maximal build-up time will yield a solution of lower daughter nuclide concentration. As a result of this elution program, the dilute daughter nuclide solution produced directly from the generator could not be used in many applications. So the radiochemical concentrating process following elution/separation of the generator should be applied to increase the concentration of separated/eluted daughter nuclide to form a suitable final ready-to-use solution. It is clear that the combination of the post-elution concentration process and the methods of build-up time optimisation and early elution schedule is the most suitable way to operate the generator effectively on the basis of economic use and purposely suitable quality of produced daughter radionuclide. This statement is justified with the result obtained from the utilization of ULTRALUTE^®^^99m^Tc concentrator device (giving a final concentrated ^99m^Tc-solution of 1.0 mL volume) to concentrate the ^99m^Tc-solution eluted in 10 mL saline solution from Gentech (ANSTO) ^99m^Tc-generator. The experimental results reported in Table 2 using a 525 mCi (~20 GBq) generator as an example confirmed that the concentration and the yield of ^99m^Tc solution eluted with a 6-h elution-schedule (Early Elution Schedule Method) is much better than that achieved with the elution performed at maximal build up time of the generator.

By combining three methods mentioned above (Build-up time optimisation method, Early elution schedule, and Post-elution concentration process), the effective utilisation of the ^68^Ge/^68^Ga generator was justified with the experimental results reported in Table 3, which were obtained by using RADIGIS-^68^Ga Radioisotope generator integrated system loaded with 18 mCi ^68^Ge-activity described in Section 3.2. The graphs presented in Figure 6 with a practical optimal build-up time *t_opt(t)_* = 2.0 h (referred to the theoretical value *t_opt(t)_* = 2.063 h reported in Table 1 for ^68^Ge/^68^Ga pair) were used for this experiment.

**Table 2:** Effectiveness of ^99m^Tc elution performed with an early elution schedule supported by a radioisotope concentrator in comparison with that normally eluted at the time point of maximal ^99m^Tc-build-up; Generator activity at calibration day (day 1, 8:00 am) is 525 mCi ^99^Mo or 459.4 mCi ^99m^Tc; Solvent is 10 mL saline; Generator is coupled with ULTRALUTE^®^ concentrator device; Final concentrated ^99m^Tc solution volume is 1.0 mL; ^99m^Tc–production efficiency in each elution: 85%–90%.

Elution Time		8 am	Day 1	Day 3	Day 6
Early elution schedule of 6 h ^99m^Tc-buidup time (4 elutions/day) performed with support of the radioisotope concentrator ULTRALUTE^®^	^99m^Tc-concentration (mCi/mL)	410	200–155	120–95	57–44
	Total yield of generator elutions per day (mCi)	415.1	683.5	420.0	197.1
Elution at maximal ^99m^Tc-buildup time (*t*_max_ = 22.86 h) (One elution/day) ^99m^Tc-elution efficiency: >95%	^99m^Tc-concentration (mCi/mL)	41.5	31.5	19.4	9.1
	Total yield of generator elution per day (mCi)	415.1	315.0	194.0	91.1

**Table 3:** Effectiveness of ^68^Ga elution performed by the combination of Build-up time optimisation and Early elution schedule method supported by an integrated elution-purification-concentration process (RADIGIS-^68^Ga system) in comparison with that normally eluted at the time point of maximal ^99m^Tc-build-up. (Generator activity at calibration day (day 1, 8:00 am) is 18.0 mCi ^68^Ge or 18.0 mCi ^68^Ga; Solvent for generator elution is 5 mL 0.1 M HCl solution; Final purified and concentrated ^68^Ga solution volume is 0.75 mL); ^68^Ga–elution efficiency: >95%.

Elution Time		8 am	10 am	12 am	14 pm	16 pm	18 pm	20 pm	Total ^68^Ga-Activity Produced
Early elution schedule of 2.0 h ^68^Ga -buid-up time (6 elutions/day) performed with support of RADIGIS-^68^Ga system	^68^Ga-concentration (mCi/mL)	22.8	16.5	16.4	16.5	16.6	16.4	16.4	-
	The yield of generator elution at 2-h build-up time (mCi)	17.1	12.4	12.3	12.4	12.5	12.3	12.3	91.3
Elution at maximal ^68^Ga-build-up time without concentrating process (* An elution at 12 h (instead of *t*_max_ = 14.1h) build-up time applied with an error of 5%)	^68^Ga-concentration (mCi/mL)	3.4	-	-	-	-	-	3.3	-
	Total yield of generator elution at maximal ^68^Ga-build-up time, mCi	17.1	-	-	-	-	-	* 16.8	33.9

The experimental results reported in Table 3 confirm that the concentration and the yield of ^68^Ga solution eluted with an optimal 2-h elution-schedule (“Early Elution Schedule” combined with “Build-up time Optimisation”) are much better than that achieved with the elution performed at maximal build up time. 

This elution plan offers cost-effective utilisation of ^68^Ge/^68^Ga generator system because the total useful ^68^Ga radioactivity gathered in 6 consecutive elutions (overall time period is 12 h) is 3 times higher (*R_y_* = 3) than that eluted once at time *t*_max_ = 14.1 h (The significant difference between practical value *R_y_* = 3 and theoretical value *R_y_* = 5 is coming from the over-evaluation caused by an overnight time overlap). Another advantage of consecutive elution performed at the optimal 2-h build-up time is the improvement in the quality of ^68^Ga solution in terms of reduction of Zn content in the ^68^Ga-eluate. An amount of 3.38 × 10^−3^ nmol ^68^Zn was evaluated at the optimal 2-h build-up time compared with the value of 0.05 nanomoles at the maximum ^68^Ga build-up time *t*_max_ = 14.1 h for the 18 mCi ^68^Ge/^68^Ga generator system.

### 4.3. Effective Control of Radionuclidic Purity: Relationship between Detection Limit, Required Radionuclidic Impurity Limit, Measurement Certainty for the Optimisation of Decay Time and QC Sample Radioactivity Used for Post-Delivery Quality Control, Quality Control of ^68^Ga Solution as a Case (Referred to Section 2.3)

**Figure 13:**
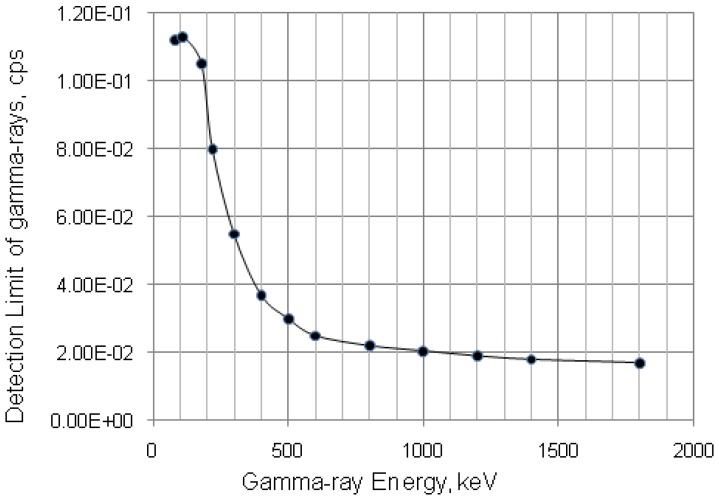
Detection limits of photo peaks, *L_D_* (not corrected with detector counting efficiency and gamma ray yield) evaluated from a background spectrum of blank sample measured for 36,000 s on HP Ge detector coupled ORTEC gamma-ray spectrometer.

As described in Section 2.3, the optimisation of decay time and QC sample radioactivity for the effective control of radionuclidic purity of daughter nuclide solution is determined by both the regulatory parameter *L* (required radionuclidic impurity limit) and technical parameters such as *L_D_* (detection limit) and *R* (measurement certainty). *L_D_* value depends on the count base line of gamma-ray energy-spectrum (specifically, on the background counts of photo-peaks of interest). The result of *L_D_* value determination using ORTEC gamma-ray spectrometer mentioned above is shown in Figure 13. This result is used in the optimisation of decay time and QC sample radioactivity based on Equations (31)–(41) in Section 2.3 for effective radionuclidic purity of control of the daughter nuclide solutions produced from different radionuclide generators in our laboratory. 

*^68^Ga-solution*: To demonstrate the effectiveness of optimisation methods developed in this paper, the control of ^68^Ge-impurity of ^68^Ga-solution which is produced from RADIGIS-^68^Ga system is shown below. ^68^Ga (*T*_1/2_ = 68 min.), which is a positron emitter used for PET imaging in nuclear medicine, is produced from ^68^Ge/^68^Ga radionuclide generator. The main impure radionuclide in ^68^Ga solution is ^68^Ge (*T*_1/2_ = 271 days) which is a parent nuclide of ^68^Ga. ^68^Ge is an electron capture radioisotope with low energy X-ray radiation. ^68^Ge contaminates ^68^Ga product solution due to its breakthrough during the elution of ^68^Ga from the generator column on which the parent nuclide ^68^Ge is immobilized. The ^68^Ge breakthrough in ^68^Ga eluate was determined based on the counts of the 511-keV photopeak of ^68^Ga activity which is in radioactive equilibrium with impure ^68^Ge activity. 

Because the majority of activity in the product eluate is ^68^Ga activity which is not in equilibrium with ^68^Ge activity at the sampling time, for the certain measurement of impure ^68^Ge activity of the ^68^Ga-eluate sample, this sample should be left for a given time so as the majority of non-equilibrium ^68^Ga activity decays to a reasonable degree to minimize the influence of the non-equilibrium ^68^Ga activity of ^68^Ga eluate sample. A relationship between the measurement certainty *R*, decay time *t* and required ^68^Ge breakthrough (impurity) limit *L* is established as follows:

**Figure 14:**
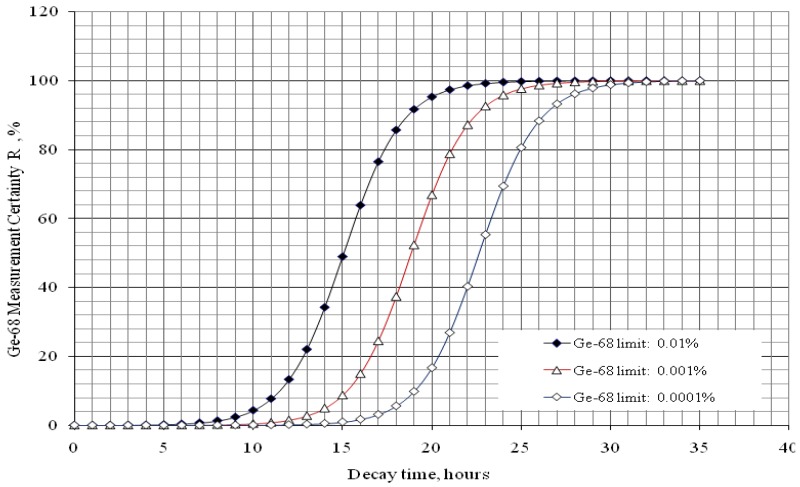
Certainty parameter R *versus* decay time t of the ^68^Ge impure radionuclide in ^68^Ga radioisotope product at different required radio-nuclidic impurity limits *L*. Poisson distribution 95% confidence interval, 
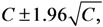
 with error < 8.77% for count numbers > 500; R(%) = 100 × (Activity of ^68^Ge/(Activity of ^68^Ge + Activity of ^68^Ga).

Detection limit of ^68^Ge measured at photo peak 511 keV with a blank sample using ORTEC gamma ray spectrometer mentioned above is *L_D_* = 0.055 × 10^−6^ μCi or 2.035 Bqs ( using the data shown in Figure 13 corrected with detector counting efficiency).

With a given certainty of measurement *R* = 98% and a required ^68^Ge breakthrough (impurity) limit *L* = 10^−3^% of the activity of the ^68^Ga eluate as stated in literature EANM-Monograph-2464-2011 (18), Table 4 and Figure 14 (which plotted from Equation (31b) show that a 25.2 h decay time is required for the ^68^Ga eluate sample to be left to decay before starting a reliable gamma-ray spectrometric ^68^Ge activity measurement. 

The minimal radioactivity *A*_0,*P*_ of the ^68^Ga eluate sample taken out from ^68^Ga product solution at the sampling time, which is used for the measurement of ^68^Ge breakthrough activity after decaying for 25.2 h, is calculated using Equation (34) which is re-written for ^68^Ge/^68^Ga nuclide pair as follows:

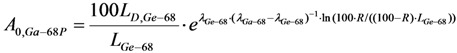


The *A*_0,*P*_ calculation given above ensures an insignificant interference for the ^68^Ge breakthrough (impurity) activity measurement of the ^68^Ga eluate sample with respect to the justified values of *R* and *L* required by regulatory compliance. Based on the assessment method described above, the ^68^Ga eluate sample of >117.55 μCi activity was justified for a reliable measurement of ^68^Ge breakthrough activity after the sample decay for a time period *t* = 25.2 h. The measurement was performed using ORTEC gamma ray spectrometer with counting time >30 min. The ^68^Ge breakthrough activity in the ^68^Ga eluate eluted from our generators was <10^−3^%. This value complies with requirement clarified in EANM-Monograph-2464-2011 [21].

**Table 4:** ^68^Ga product samples and relevant parameters of the designed protocol for gamma ray spectrum analysis of ^68^Ge impure radionuclide. Gamma ray spectrum was measured with a dead time of 1%; Detection limits measured with a blank sample; Poisson distribution 95% confidence interval, 
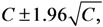
 with error < 8.77% for count numbers > 500. *R*(%) = 100 × (*Activity of ^68^Ge* / (*Activity of ^68^Ge* + *Activity of ^68^Ga*).

Required radionuclidic impurity limit *L*, %	Required certainty degree *R*, %	Required decay time *t*, hours	Activity of ^68^Ga–sample taken out from ^68^Ga–product solution at the product delivery time point (Measurement: 640 min counting for collecting 1000 counts; Error 6.2%)	Activity of ^68^Ga–sample taken out from ^68^Ga–product solution at the product delivery time point (Measurement: 30 min counting for collecting 1000 counts; Error 6.2%)
0.01	98	21.43	0.55 μCi	11.74 μCi
0.001	98	25.20	5.51 μCi	117.55 μCi
0.0001	98	28.96	55.17 μCi	1176.96 μCi
Note: Counting rate (cps) at 511 keV = (Detection limit × Gamma ray yield × Counting efficiency) = 2.035 Bqs × 1.78 × 0.0072 = 0.026 cps. Counting time, *t* = 1000 counts/0.026 cps = 640 min				Note: Proportional increase in sample activity can be used to reduce measurement time

*^99m^Tc-solution*: The ^99m^Tc-solution produced from the ^99m^Tc-generators may contain the ^99^Mo-parent nuclide and a number of extraneous contaminants such as ^103^Ru, ^131^I, ^132^Te, ^99^Zr, ^124^Sb, ^134^Cs, ^89^Sr, ^90^Sr, and ^86^Rb. The ^99^Mo-contamination is measured by the “shielding” method as mentioned in Section 2.3. The 760 keV and 780 keV photons of the ^99^Mo contamination in ^99m^Tc-solution housed in a 6 mm thick lead pot are measured in a dose calibrator. Other gamma-emitting nuclide contaminations with the contamination limits (*L* values) required by USP-32 [22] are checked by the “decay” method as mentioned in Section 2.3.2. (The β^−^ and α-emitting nuclides are not more than 0.01% of ^99m^Tc activity and 0.001nCi/mCi^99m^Tc, respectively). The γ-emitting radionuclide contaminations can be checked by a gamma-ray spectrometer after allowing ^99m^Tc to decay to a certain extent. For demonstration of the “decay” method developed in this paper, the example of the optimisation of ^131^I contamination determination is detailed as follows: 

The ^131^I contamination limit (*L*) in the ^99m^Tc-solution required by USP-32 is L = 0.05 μCi/mCi 0.05 Bq/kBq) ^99m^Tc or in percentage *L* = 0.005%.

The ORTEC multichannel gamma-ray spectrometer coupled with HP-Ge detector is used. As experienced, the dead time of the spectrum acquisition using this system is 1% for the ^99m^Tc-sample activity *A_t_*_,*P*_ = 2 μCi ^99m^Tc.

Referred to Section 2.3.2, Equations (35)–(37) are used for calculate the optimal decay time *t* and the minimum activity of the QC sample of ^99m^Tc solution taken at the ^99m^Tc-product delivery time for a gamma-ray spectrometric measurement after allowing ^99m^Tc to decay to the value *A_t_*_,*P*_ = 2 μCi ^99m^Tc. The *L_D_* value for the 0.365 MeV photon of the ^131^I nuclide, which is corrected with a counting efficiency ɛ = 0.007 and gamma yield *y* = 82%, is *L_D_* = 8.7cps (the uncorrected value shown in Figure 13 is *L_D_* = 5 × 10^−2^ cps):

*λ*_I-131_ = 9.98 × 10^−7^ sec^−1^; *λ*_Tc-99m_ = 3.19 × 10^−5^ sec^−1^

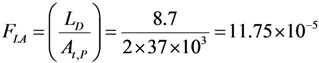


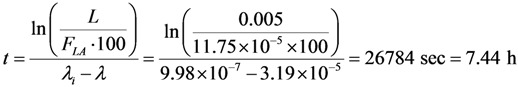


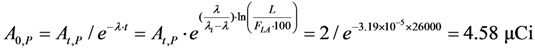



Based on the obtained results, it is stated that the ^99m^Tc-sample of ~5 μCi activity is suitable for the determination of ^131^I contamination by gamma-ray spectrometry after allowing ^99m^Tc-sample to decay for a time period of 7.44 h. The same process will be performed to assess the optimal decay time and the minimal activity of the QC sample of ^99m^Tc-solution for other radionuclide contaminations of the ^99m^Tc-solution. Finally, among the assessed samples, the largest activity sample allowed to decay for a corresponding longest decay time will be suitable for a gamma-ray spectrometric measurement of different radionuclide contaminations of the ^99m^Tc-solution.

## 5. Conclusions

Optimisation method of daughter nuclide build-up *versus* stand-by time and specific activity using mean progress functions were successfully developed for increasing the performance of radionuclide generators. A new characteristic parameter (optimal build-up time *t_opt_*_(*t*)_) of the formation-decay kinetics of parent/daughter nuclide systems was found and effectively used in the practice of generator production and utilisation. The combination of the post-elution concentration process, the build-up time optimisation method, and the “early elution schedule” method is the most suitable way to operate the generator effectively on the basis of economic use and improvement of purposely suitable quality of the produced daughter radionuclide, thus there is an increase in the effectiveness of parent/daughter nuclide utilisation. The relationships between gamma ray spectrometric detection limit, required limit of impure radionuclide activity and its measurement certainty with respect to optimising decay/measurement time and product sample activity used for radionuclidic purity quality control were studied and formulated in the format of mathematical equations which are useful for the spectrometric measurement of very low activity of impure radionuclide contamination in a radioisotope product of much higher activity used in molecular PET and SPECT imaging and monoclonal antibody/peptide-targeted radiotherapy. 
